# Contralateral Effects of Unilateral Strength and Skill Training: Modified Delphi Consensus to Establish Key Aspects of Cross-Education

**DOI:** 10.1007/s40279-020-01377-7

**Published:** 2020-11-11

**Authors:** A. Manca, T. Hortobágyi, T. J. Carroll, R. M. Enoka, J. P. Farthing, S. C. Gandevia, D. J. Kidgell, J. L. Taylor, F. Deriu

**Affiliations:** 1 Department of Biomedical Sciences, University of Sassari, Viale San Pietro 43/b, 07100 Sassari, Italy; 2 Center for Human Movement Sciences, University Medical Center Groningen, University of Groningen, Groningen, The Netherlands; 3 Centre for Sensorimotor Performance, School of Human Movement and Nutrition Sciences, The University of Queensland, Brisbane, QLD Australia; 4 Department of Integrative Physiology, University of Colorado Boulder, Boulder, USA; 5 University of Saskatchewan College of Kinesiology, Saskatoon, SK Canada; 6 Neuroscience Research Australia (NeuRA), The University of New South Wales, Sydney, Australia; 7 Department of Physiotherapy, School of Primary and Allied Health Care, Faculty of Medicine, Nursing and Health Science, Monash University, Melbourne, Australia; 8 School of Medical and Health Sciences, Edit Cowan University, Joondalup, Australia

## Abstract

**Background:**

Cross-education refers to increased motor output (i.e., force generation, skill) of the opposite, untrained limb following a period of unilateral exercise training. Despite extensive research, several aspects of the transfer phenomenon remain controversial.

**Methods:**

A modified two-round Delphi online survey was conducted among international experts to reach consensus on terminology, methodology, mechanisms of action, and translational potential of cross-education, and to provide a framework for future research.

**Results:**

Through purposive sampling of the literature, we identified 56 noted experts in the field, of whom 32 completed the survey, and reached consensus (75% threshold) on 17 out of 27 items.

**Conclusion:**

Our consensus-based recommendations for future studies are that (1) the term ‘cross-education’ should be adopted to refer to the transfer phenomenon, also specifying if transfer of strength or skill is meant; (2) functional magnetic resonance imaging, short-interval intracortical inhibition and interhemispheric inhibition appear to be promising tools to study the mechanisms of transfer; (3) strategies which maximize cross-education, such as high-intensity training, eccentric contractions, and mirror illusion, seem worth being included in the intervention plan; (4) study protocols should be designed to include at least 13–18 sessions or 4–6 weeks to produce functionally meaningful transfer of strength, and (5) cross-education could be considered as an adjuvant treatment particularly for unilateral orthopedic conditions and sports injuries. Additionally, a clear gap in views emerged between the research field and the purely clinical field.

The present consensus statement clarifies relevant aspects of cross-education including neurophysiological, neuroanatomical, and methodological characteristics of the transfer phenomenon, and provides guidance on how to improve the quality and usability of future cross-education studies.

**Electronic supplementary material:**

The online version of this article (10.1007/s40279-020-01377-7) contains supplementary material, which is available to authorized users.

## Key Points

**Table:** 

Cross-education refers to the increased motor output (i.e., force generation, skill) of the opposite, untrained limb following a period of unilateral exercise training. Despite extensive research, several aspects of the transfer phenomenon remain controversial.
The present consensus statement clarifies relevant aspects of cross-education including neurophysiological, neuroanatomical, and methodological characteristics of the transfer phenomenon.
A clear gap in views emerged between the research and the purely clinical fields.
Guidance from leading experts in the field is provided on how to improve the quality and usability of future cross-education studies.

## Introduction

An imposing body of evidence obtained under a variety of experimental conditions has demonstrated that unilateral motor practice improves the motor output in both the exercised and the unexercised homologous muscles [1, 2]. A wide range of terms have been used to describe this phenomenon, such as cross-education, interlimb transfer, contralateral effect, contralateral transfer, cross-transfer, and bilateral transfer, etc. The most frequently used term is cross-education. However, this term is typically used when applied to reference to strength training and fails to denote its application to skill transfer [3]. First reported more than one century ago in the psychomotor literature [4–6], this well-known phenomenon continues to attract the attention of both basic and applied scientists who investigate its physiological underpinnings and explore its potential to treat unilateral impairments. The findings of more than 100 individual studies on strength and skill transfer have been summarized in narrative [1, 7–13], systematic [14, 15], and meta-analytic reviews [16–20]. Overall, the aggregate data confirm the robustness of the phenomenon and identifies contexts in which the transfer is particularly consistent among the studies, e.g., voluntary dynamic contractions, eccentric contractions, electrical stimulation, whole-body vibration, and mirror feedback training) [21]. The transfer effect is generally considered muscle-specific, mainly involving the contralateral homologous muscles, even though a small spatially distributed effect to at least synergists can occur [22]. Also, cross-education produced by one type of muscle contraction is specific, because the cross-education effect is much less when tested in another type of muscle contraction [21]. However, the effects can also be nonspecific (e.g., training the shoulder abductors on one side can increase the motor output of the contralateral lateral trunk flexors). The magnitude of cross-education seems to decrease with age [23]. In addition, cross-education has been demonstrated not only in the neurologically intact but also in patients with neurologic disorders, such as stroke [24, 25] and multiple sclerosis [26].

Although many aspects of the contralateral effects of unilateral motor practice are established, there is much heterogeneity in the data, especially on the neural mechanisms mediating the transfer of strength and skill, with the corpus callosum being considered to play an essential role in the transfer [9].

Similarly, the translational relevance of contralateral approaches to rehabilitation remains controversial among basic science researchers and clinicians. Moreover, the absence of a consensus on terminology seems to have contributed to fragmentation of the literature on this topic. Through this paper, which is a part of a broader scholarly initiative that gathers leading experts in the field, we aimed at establishing consensus on terminology, methodology, mechanisms of action, and translational potential of cross-education. We also intend for this paper to provide a framework for future research on the topic. Here, we present the details of this modified two-round Delphi consensus study.

## Methods

The Delphi technique is a structured method to elicit opinions on given questions from a group of experts and stakeholders [27], and used increasingly in research, health, and medicine as a tool to address issues and develop consensual guidance on best practice.

In accordance with the recommendations on Conducting and REporting DElphi Studies (CREDES) [28], we planned a process characterized by the involvement of experts with diverse backgrounds (i.e., physiologists, sports scientists, neurologists, physiotherapists) and irrespective of geographical location. The participants respond anonymously to a questionnaire that sequentially incorporates feedback into a refined survey. The process is iterative in nature and, unlike regular one-round surveys, it comprises two or more rounds of enquiry. Following each round, averaged responses from the group are summarized in a report provided to each respondent, allowing them to reconsider their own views on the topic. The whole process of consensus building is conducted through electronic survey. To comply with CREDES recommendations, the above features were incorporated in the present Delphi process.

### Delphi Survey Questions: Contralateral Effects of Unilateral Motor Practice

The Delphi survey comprised 29 questions that probed five themes: (1) terminology and definition of the phenomenon (questions 1–4); (2) theoretical explanatory models (questions 5–8); (3) neurophysiological and neuroanatomical techniques and evidence (questions 9–14); (4) practical aspects regarding the administration of contralateral protocols (questions 15–21); (5) clinical relevance, application, and barriers (questions 22–27). Questions 28 and 29 asked the participants to report demographic and background information.

Questions for the first and second themes emerged from works that identified salient features of the phenomenon and its central and peripheral mechanisms [7, 8]. Questions for the third theme were informed by both seminal works [29–32] and the most recent syntheses of the available evidence [12, 15, 20]. Questions on the practical aspects and on the clinical outreach (themes 4 and 5) were developed iteratively by the members of the research team (AM, FD, TH) and based on the few clinically oriented reviews that are available [10, 13, 14, 33].

The survey was reviewed and pilot-tested by an external board of six ‘core experts’ (TC, RE, JF, SG, DK, and JT). Feedback received during review and piloting was incorporated into the survey.

### The Delphi Process

An online software service [SurveyMonkey http:/survey-monkey.com] was used to deliver rounds 1 and 2 of the Delphi survey electronically. Identified experts were invited to participate via an email that included key information about the study, its purpose, how it would inform consensus on terminology, methodology, mechanisms of action, and translational aspects of contralateral transfer, and directions for future research. Rounds were available online for 4 weeks each. Three reminders were sent to participants on days 7, 14, and 21.

Participants were asked to respond to each question on a 5-point Likert scale (e.g., 1, strongly disagree; 2, disagree; 3, neutral; 4, agree; 5, strongly agree), and by ranking items in 4 out of 29 questions (13.8%; questions 11–14). A cut-off of 75% agreement was chosen as the consensus threshold based on the findings of a systematic review of Delphi studies [34]. Accordingly, we considered consensus to be reached if at least 75% of respondents scored the question 4–5 (positive consensus towards agreement) or 1–2 (negative consensus towards disagreement) on the 5-point Likert scale. For ranking questions, we analyzed the distribution of the response frequencies and considered only the first three in rank, based on the number of preferences received.

#### Round 1

Participants answered the 29 questions via the online survey (Supplementary File 1). We asked them for any additional comments/insights which they wanted to provide using free-text boxes.

#### Round 2

Based on the results and comments from round 1, the research team and the panel of core experts agreed to remove questions for which consensus had been reached, delete or modify unclear questions and sub-items, and include additional questions and sub-items suggested by participants. As a criterion for eliminating questions or sub-items, the research team and the board of core experts agreed on setting a cut-off threshold at < 50%. Questions that did not reach at least the 50% threshold were, therefore, discarded from the survey and not resubmitted in round 2.

We then invited participants to complete round 2 of the Delphi process (Supplementary File 1). In the invitation, they were provided with aggregate, de-identified results from round 1 in the form of a narrative summary of the survey results, graphical representations of the data, as well as percentages and response frequencies. As in round 1, participants were allowed to provide comments and insights using free-text boxes in round 2.

### Participants

Through literature scan of four biomedical databases (PubMed/Medline, Scopus, Web of Science, SPORTDiscus) and using common keywords specifically related to the phenomenon (cross-education, contralateral effect, contralateral training/exercise/practice, unilateral training/exercise/practice, interlimb transfer, contralateral transfer, cross-transfer, bilateral transfer, strength transfer, skill transfer), we identified 137 authors who published at least one article on the topic (as of July 31, 2019). Of these individuals, 56 had authored at least two articles with a prominent role (first or second or last or corresponding author), with 38 of them authoring at least three. The more conservative cut-off was agreed upon by the panel of experts (8 out of 9, 88.9%) as a criterion to qualify authors for inclusion and invitation. After extracting contact information, electronic invitations were sent to 56 authors.

### Data Analysis

Discrete variables in the form of counts/proportions/percentages are reported.

## Results

### Participation by Round

Of the 56 invitation emails sent for round 1 (October 29 to November 28, 2019) of the Delphi process, 34 invitees (60.7%) completed the 29-question survey. In round 2 (January 23 to February 22, 2020), 32 of round-1 respondents completed the restructured 18-question survey. In both rounds, participants provided detailed comments in the text boxes to support their responses or including additional comments on the topic, in general, or specific to a given question/sub-item. Responses were received from a minimum of 28 to a maximum of 33 participants in round 1 (82–100%), and rose to 30–32 (94–97%) in round 2. Data were ultimately analyzed from the 32 respondents who completed both rounds 1 and 2.

Table 1 details the respondents’ characteristics. There was an international representation, including participants from 12 countries. Diversity in background was also present, with 18 out 32 (56%) being sport scientists, 6 medical doctors (19%), 5 physiotherapists (16%), 2 neuroscientists (6%), and 1 biologist (3%). Clinicians counted for 34% (11/32) of the cohort.

**Table 1:** Respondents’ characteristics (*n* = 32)

	*n*	(%)
Background		
Biologist	1	3
Medical doctor	6	19
Neuroscientist	2	6
Physiotherapist	5	16
Sport scientist	18	56
Research engagement*		
Applied science	26	72
Basic science	17	53
No research engagement	2	6
Geographical location		
Australia	11	34
Belgium	1	3
Canada	5	16
Ireland	1	3
Italy	2	6
Japan	1	3
The Netherlands	2	6
New Zealand	3	9
Spain	1	3
Switzerland	1	3
United Kingdom	3	9
United States	1	3

*Percent values do not add up to 100, since some respondents identified themselves as part of more than one category

Table 2 summarizes all items reaching consensus and the round at which consensus was reached. Next, we describe the survey results for the four main themes identified.

**Table 2:** Delphi items that reached consensus

Delphi items	Round	*n* (%)
1. Cross-education as the term to refer to the transfer phenomenon	1	24/30 (80)
2. Important elements to be part of the definition		
Homologous muscles	2	24/30 (80)
3. Theoretical models to explain the phenomenon: bilateral access (a.k.a. ‘callosal access’) or cross-activation (a.k.a. ‘spillover’)		
Both models involved for skill transfer	2	23/30 (77)
4. Involvement of the mirror-neuron system in skill transfer	1	21/28 (75)
5. TMS-based parameters to be included in the ideal neurophysiologic assessment of the phenomenon		
Short-interval intracortical inhibition	1	21/26 (81)
Interhemispheric inhibition	2	22/29 (76)
6. Mechanisms most likely to mediate the phenomenon		
Reduced interhemispheric inhibition for strength transfer	1	Ranked 1st
Reduced interhemispheric inhibition for skill transfer	1	Ranked 1st
7. Techniques most likely to capture adaptations to unilateral training		
Functional magnetic resonance imaging	1	Ranked 1st
8. Primary motor cortex as the central nervous system site most likely to mediate/contribute to the phenomenon	1	Ranked 1st
9. Investigating the role of muscular mechanisms with modern technologies	2	26/32 (81)
10. Strategies to maximize the magnitude of the transfer of strength and/or skills		
Eccentric actions	2	27/32 (84)
High-intensity training	1	27/30 (90)
Mirror illusion	2	28/32 (88)
11.’13–18 sessions’ as adequate dose of training sessions* to obtain significant contralateral gains in strength	2	22/29 (76)
12. Need for future investigations on the time-course of the crossed adaptations to unilateral training	1	26/29 (90)
13. Clinical utility of the transfer		
Strength transfer	1	28/31 (90)
Skill transfer	1	30/31 (97)
14. Clinical scenarios that may benefit from the phenomenon		
Orthopedic conditions	1	23/28 (82)
Sport injuries	1	28/30 (93)
15. Potentials barriers to the clinical employment of contralateral approaches		
Inadequate scholars’ and clinicians’ education/training	1	26/30 (87)
Lack of studies assessing the clinical importance and meaningfulness of the crossed gains (i.e., minimal important difference)	1	26/30 (87)
16. Regarding the warning that contralateral training may enhance interhemispheric imbalance and strength/skill asymmetry, asymmetry is less important if there are benefits for the more-affected limb**	1	19/25 (76)
17. Need to develop a road map (i.e., scoping review) to critically appraise the clinical potential of the phenomenon	1	28/32 (88)

*TMS* transcranial magnetic stimulation, *a.k.a.* ‘also known as’

*With a standard frequency of 3 sessions/week. Questions 6–8 were presented as ranking items, with consensus reported in the table only for the item first in rank

**Item framed with reference to unilateral impairment of neurological origin, mainly stroke

#### Terminology and Definition

At round 1 consensus was reached on the term ‘cross-education’ to indicate the transfer effect (24/30, 80%). Six authors commented that the term should always be context-specific by clearly stating if a transfer of strength or skill is meant (i.e., cross-education of strength vs. cross-education of skill).

There was no consensus in either round 1 (53%) or round 2 (47%) on the need to update the current definition of the phenomenon, i.e., the increase in muscle strength and/or motor skills in the opposite, untrained limb following a period of unilateral exercise training. Nevertheless, in round 2, consensus was reached (24/30, 80%) for the inclusion of ‘homologous muscles’ in the definition.

#### Theoretical Models

Two questions assessed the degree of agreement on two theoretical models commonly used to explain the transfer of strength and, separately, skill: ‘bilateral access’ (aka ‘callosal access’) and ‘cross-activation’ (aka ‘spillover’). In round 2 and only for skill transfer, consensus was reached on ‘both models involved’ (23/30, 77%).

Consensus was also reached (round 1, 21/28, 75%) on the mirror-neuron system as a possible contributor to the transfer of skill but not of strength (53% in round 1; 61% in round 2).

No consensus was reached in the two rounds on the relevance of priming the ipsilateral primary motor cortex to augment the transfer of strength (55% in round 1; 57% in round 2) nor skill (59% in round 1; 63% in round 2).

#### Neurophysiological and Neuroanatomical Evidence

**Fig. 1:**
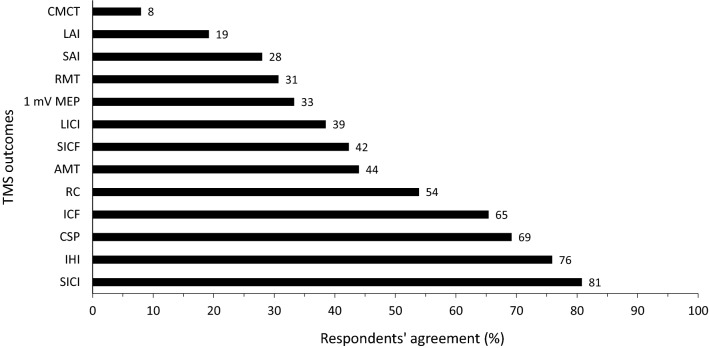
Respondents’ agreement on transcranial magnetic stimulation outcomes proposed in the survey. *1 mV MEP* 1-Millivolt motor-evoked potential, *AMT* Active motor threshold, *CMCT* Central motor conduction time, *CSP* Cortical silent period, *ICF* Intracortical facilitation, *IHI* Interhemispheric inhibition, *LAI* Long-latency afferent inhibition, *LICI* Long-interval intra-cortical inhibition, *RC* Recruitment curve, *RMT* Resting motor threshold, *SAI* Short-latency afferent inhibition, *SICF* Short-interval intracortical facilitation, *SICI* Short-interval intra-cortical inhibition

Thirteen parameters measured by transcranial magnetic stimulation (TMS) were evaluated (Fig. 1). Of these, short-interval intracortical inhibition (SICI) was identified by consensus in round 1 (21/26, 81%) as an important parameter to include in the ideal neurophysiological assessment of the transfer. In round 2, consensus was also reached for interhemispheric inhibition (IHI, 22/29, 76%). Relatedly, a reduction in IHI was ranked as the most likely mechanism to accompany the transfer of both strength and skill, with consensus reached in round 1 for both contexts. In round 1, reduced SICI was ranked in the second place for strength transfer, and third for skill transfer.

In round 1, functional magnetic resonance imaging (fMRI) was ranked as the most likely technique to capture the adaptations, followed by TMS-based assessments.

When asked in round 1 to rank the site within the central nervous system most likely to be associated with the phenomenon, participants listed the ‘primary motor cortex’ in first place followed by ‘supplementary motor area’, ‘primary somatosensory area’, and ‘dorsal premotor cortex’.

Participants also agreed by consensus (round 1, 26/32, 81%) on the need to employ modern technologies in future research to examine the role played by muscular mechanisms in the contralateral transfer of performance.

Regarding the efficacy of specific strategies that have been reported to enhance the magnitude of the transfer, ‘high-intensity training’ reached consensus in round 1 (27/30, 90%), whereas consensus for ‘mirror illusion’ (28/32, 88%) and ‘eccentric actions’ (27/32, 84%) was reached in round 2.

No consensus was reached for the direction of the transfer (i.e., dominant to non-dominant, or vice versa), for either the upper or lower limbs (for all items, less than 50% at round 1).

For the duration of unilateral practice protocols, participants deemed ‘13–18 sessions’ (with a standard frequency of 3 sessions/week) as an adequate time frame to obtain a significant transfer of strength (consensus reached in round 2: 22/29, 76%), whereas no consensus was reached for skill transfer for the ‘7–12 sessions’ time frame, which obtained the largest number of preferences (56% in round 1; 68% in round 2). Accordingly, future research to investigate the time-course of the transfer was deemed ‘definitely worthy’ by consensus in round 1 (26/29, 90%).

Despite the well-known difference in strength between men and women, no consensus was reached on the proposal that studies on unilateral strength training should examine sex differences (69% in round 1; 65% in round 2).

#### Clinical Relevance, Application, and Barriers

Because contralateral approaches (i.e., training the sound/less-affected limb to obtain crossed motor improvements in the untrained, more-affected side) have been advocated for the management of unilateral motor impairment of different pathological origin, the participants were asked whether the phenomenon had potential clinical utility. Consensus on utility was reached in round 1 both for strength (28/31, 90%) and skill transfer (30/31, 97%). When asked to judge a range of clinical scenarios, participants agreed in round 1 on the potential utility of the phenomenon for orthopedic conditions (23/28, 82%) and sports injuries (28/30, 93%). No consensus was achieved for central neurological conditions in either round 1 (59%) or round 2 (67%).

When asked about the warning that contralateral training may enhance interhemispheric imbalance and strength asymmetry, 76% of the respondents (round 1, 19/25) agreed that ‘asymmetry is less important if there are benefits for the more affected limb’.

With regard to factors that may act as potential barriers to the clinical employment of contralateral approaches, consensus was reached in round 1 for ‘Inadequate scholars’ and clinicians’ education/training’ (26/30, 87%), and ‘lack of studies assessing the clinical importance and meaningfulness of the crossed gains’ (26/30, 87%).

Finally, there was a round-1 consensus concerning the need to develop a road map (i.e., scoping review) and critically evaluate the clinical potential of the phenomenon (28/32, 88%).

## Discussion

We conducted a two-round Delphi process with the aim of reaching consensus on five themes ranging from terminology and definition to neurophysiological and neuroanatomical features and, ultimately, the clinical relevance of the transfer phenomenon. By building consensus, we intended to establish a common platform to streamline future research on the mechanistic underpinnings as well as the clinical application of cross-education.

We have reached consensus on 18 of the 27 (67%) proposed questions (questions 28 and 29 were related to demographic and background characteristics of the invitees). Experts reached consensus quickly concerning the labeling of the phenomenon as ‘cross-education’. The majority of the participants (80%) considered the term as a “brand” name that clearly describes to the phenomenon. Experts concluded that any change in terminology by employing several other operational terms and keywords would result in fragmentation of the literature into parallel subsets of knowledge, thus hindering its unitary appraisal, understanding, and advancement. However, we received several comments on possible limitations in the use of this term (e.g., transfer of strength vs. skill), so authors should specify if cross-education of strength or skill is meant in the article.

Consensus was reached on avenues of physiological and anatomic interest, such as mechanisms and substrates behind cross-education. In particular, regarding the methods used to capture neural adaptations in response to unilateral training, fMRI was ranked first, followed by TMS assessment. In this regard, there was agreement on including at least SICI and IHI in the ideal neurophysiological investigation. Moreover, modulation of inhibitory circuitry was reported as the main mechanism underpinning the transfer, which participants suggested mainly occurs in the ipsilateral primary motor cortex and supplementary motor area.

Training variables and methods can affect the magnitude of cross-education. Survey participants agreed by strong consensus that high-intensity training, eccentric contractions, and mirror illusion are effective and promising strategies to maximize the magnitude and translational implications of the transfer. However, consensus was not reached on the utility of alternative priming approaches such as neuromodulation by transcranial direct current stimulation, which has been explored in a few articles [35, 36]. There is currently insufficient evidence in support of using priming of the trained and transfer muscle or hemisphere.

Similarly, there was no consensus nor even a trend on the direction of the transfer (i.e., dominant to non-dominant, or vice versa), which requires further research to be integrated with previously published reports [37, 38]. This uncertainty may reflect the possibility that different features of motor adaptation transfer more effectively in different directions, which may in turn reflect hemispheric specialization of sensorimotor function [39].

For the duration of training, our consensus was that 13–18 sessions or 4–6 weeks is the shortest duration needed to produce functionally meaningful transfer of maximal voluntary force. By contrast, there was no consensus for the minimal duration for producing functionally meaningful cross-education of motor skills. Strong consensus, however, was reached on the need for future studies to integrate with previously published reports [2, 40] to evaluate the time-course of the adaptations produced by unilateral training protocols, which is relevant both for researchers and practitioners who are planning to translate cross-education into clinical scenarios.

The clinical relevance of cross-education, which has been the topic of a number of clinically oriented reviews [10, 11, 13, 14, 33], was one of the five themes of the present Delphi process, and was assessed by 5 questions (22, 23, 25–27). In round 1, > 90% of respondents agreed on the potential clinical utility of the transfer, both for strength and motor skills. Cross-education was deemed suitable to orthopedic conditions and sports injuries, which is consistent with promising data from orthopedic cohorts [41, 42], even though other studies have reported no significant value in adding cross-education to conventional rehabilitative programs [43, 44]. Unexpectedly, no consensus was reached in either round (59–67%) regarding the potential use of cross-education for treating neurological patients, despite favorable findings in stroke survivors and people with multiple sclerosis [24–26, 45–49]. This discrepancy between the results of the consensus and the literature may be partly explained by the lack of familiarity of many respondents with the neurological/neurorehabilitative literature. Indeed, the composition of the respondents’ group mainly consisted of sports scientists and sports physiotherapists, who may be more familiar with sports and orthopedic than neurological populations, thus introducing selection bias. Interestingly, this was the item receiving the largest number of comments, among which the most recurrent opinions were: “*clinical utility in models of musculoskeletal pathology*” and “*very promising or detrimental depending on the specific neurological condition and state of the CNS*”.

These results seem to reflect the general opinion that the transfer can happen through a healthy nervous system in orthopedic/sports injuries compared to what might happen in a damaged nervous system, where the processes that are thought to happen in healthy people may be disrupted.

As evidence of the ‘perceived’ clinical utility of cross-education in the management of unilateral injuries, participants strongly agreed (88%) on the need to develop a scoping review to evaluate the clinical potential of cross-education. Scoping reviews 'map' the literature on a particular topic or research area and identify key concepts, gaps in the knowledge, and types and sources of evidence to inform practice, policymaking, and research. When specific literature is heterogeneous or influenced by conceptual or methodological limitations, scoping reviews are increasingly recognized to aid the planning and commissioning of future research [50]. A scoping review on cross-education would allow to establish a common platform for researchers and clinicians and to enhance the quality and practical relevance of research in this field. With a road map for future action and with the present consensus statement obtained by bringing together the expertise, guidance, and insights of leading experts in the field, we will be better positioned to study the phenomenon of cross-education, reduce the gap in views between researchers and clinicians, and examine the potential translation into clinical practice.

## Limitations

The findings of the present Delphi process are limited by the characteristics of the selected contributors who participated in the survey, possibly subjecting the survey findings to selection bias. Although we were able to involve the majority (61%) of those scientists qualified as cross-education experts, we cannot exclude that responses from a larger number of individuals with different backgrounds may have led to different results, especially on the clinical relevance of cross-education. In this regard, some bias may have been introduced by our questioning method, which allowed participants to pick only one professional category. This may have led to the relatively low sample of respondents declaring to be clinical practitioners. Finally, while we attempted to be comprehensive in the development of the survey questions and sub-items, other questions could have been asked, so that other specific issues of cross-education may have been overlooked.

## Concluding Remarks

Based on the consensus reached, our recommendations for future studies are that (1) the term ‘cross-education’ should be adopted to refer to the transfer phenomenon, also specifying if transfer of strength or skill is meant; (2) functional magnetic resonance imaging, short-interval intracortical inhibition, and interhemispheric inhibition appear to be promising tools to study the mechanisms of transfer; (3) strategies which maximize cross-education, such as high-intensity training, eccentric contractions, and mirror illusion, seem worth being included in the intervention plan; (4) study protocols should be designed to include at least 13–18 sessions or 4–6 weeks to produce functionally meaningful transfer of strength, and (5) cross-education could be considered as an adjuvant treatment particularly for unilateral orthopedic conditions and sports injuries.

In conclusion, the Delphi process clarified several aspects of cross-education ranging from sharing a unique term to clinical potential of the phenomenon and identified neurophysiological, neuroanatomical, and methodological characteristics of cross-education, and guidance on future directions to improve the quality and usability of upcoming research on this topic.

## Electronic supplementary material

Below is the link to the electronic supplementary material.

Supplementary file1 (DOCX 80 kb)

Supplementary file2 (PDF 3187 kb)

Supplementary file3 (PDF 609 kb)
