# Supplementary material for: Contralateral Effects of Unilateral Strength and Skill Training: Modified Delphi Consensus to Establish Key Aspects of Cross-Education
Source: Sports Med. 2020 Nov 11;51(1):11–20. doi: 10.1007/s40279-020-01377-7 (PMC7806569; doi:10.1007/s40279-020-01377-7)
Supplement: Supplementary file 1 — Supplementary file1 (DOCX 80 kb) [file 40279_2020_1377_MOESM1_ESM.docx]

**Delphi process for cross-education**

**Round 1 of 2**

*If the content of some items falls outside your specific expertise, so that you would feel uncomfortable or inadequate to provide an answer, please leave the items unanswered (consensus will be calculated from responders). Please, also specify the reason/s for non-response in the comments below the item/s left blank.*

1. Cross-education (from now on: ‘the phenomenon’ or ‘unilateral training’ or ‘contralateral training’ or ‘unilateral contralateral training’) is generally defined as the increase in muscle strength and/or motor skills in the opposite, untrained limb following a period of unilateral exercise training.

Should the definition be updated or expanded?

| No update/expansion  is  required |  |  |  | Neutral |  |  |  | An  update/  expansion  is required |
| --- | --- | --- | --- | --- | --- | --- | --- | --- |
| O | O | O | O | O | O | O | O | O |

Other comments (with reasons) _______________________________________________

2. Based on your knowledge, experience and experimental evidence, please judge the importance of the following elements to be part of the definition, in case an update is deemed necessary:

|  | Not  important at all |  |  |  | Neutral |  |  |  | A very  important element |
| --- | --- | --- | --- | --- | --- | --- | --- | --- | --- |
| Homologous  muscles | O | O | O | O | O | O | O | O | O |
| Intensity of  the training | O | O | O | O | O | O | O | O | O |
| Main  putative  neural  mechanism | O | O | O | O | O | O | O | O | O |
| Neural nature | O | O | O | O | O | O | O | O | O |
| Skill  transfer | O | O | O | O | O | O | O | O | O |
| Strength  transfer | O | O | O | O | O | O | O | O | O |
| Training-  specific | O | O | O | O | O | O | O | O | O |

Other elements to be considered______________________________________________

Other comments (with reasons) _______________________________________________

3. A considerable body of knowledge has accumulated so far on the phenomenon, i.e. contralateral effects of unilateral training (both strength and skill) and associated adaptations. Most researchers currently refer to this broad topic with the term ‘cross-education’. Should this term be changed?

| No change  in name  required |  |  |  | Neutral |  |  |  | An  alternative  name  is required |
| --- | --- | --- | --- | --- | --- | --- | --- | --- |
| O | O | O | O | O | O | O | O | O |

Other comments (with reasons) _______________________________________________

4. Please, rate the following terms to indicate the phenomenon.

|  | A terrible name |  |  |  | Neutral |  |  |  | A very  brilliant  name |
| --- | --- | --- | --- | --- | --- | --- | --- | --- | --- |
| Cross-education | O | O | O | O | O | O | O | O | O |
| Contralateral  effects | O | O | O | O | O | O | O | O | O |
| Contralateral  effects of  unilateral training |  |  |  |  |  |  |  |  |  |
| Contralateral  training  effect | O | O | O | O | O | O | O | O | O |
| Cross-training | O | O | O | O | O | O | O | O | O |
| Cross-training effect | O | O | O | O | O | O | O | O | O |
| Cross-transfer | O | O | O | O | O | O | O | O | O |
| Cross-transfer  effect | O | O | O | O | O | O | O | O | O |
| Interlimb  transfer | O | O | O | O | O | O | O | O | O |

Other suggested names_________________________________________

Other comments (with reasons) _______________________________________________

5. Two theoretical models involving neural plasticity of the brain have been proposed to explain the phenomenon:

(1) the “bilateral access” (aka ‘callosal access’), which involves the development of motor engrams following unilateral movement that can be accessed not only by the trained limb, but also by the untrained limb;

(2) the “cross-activation” hypothesis (‘spillover’), which is based on the concept of unilateral contractions being driven by bilateral cortical activity in both the contralateral and ipsilateral motor cortex, producing lasting neuroplasticity in both cortices;

Based on your knowledge, experience and experimental evidence, please state your degree of agreement with each model, distinguishing between the strength and skill paradigms.

|  |  | Strongly  disagree |  |  |  | Neutral |  |  |  | Definitely  Agree |
| --- | --- | --- | --- | --- | --- | --- | --- | --- | --- | --- |
| Strength  paradigm | Bilateral  access | O | O | O | O | O | O | O | O | O |
|  | Cross-  activation | O | O | O | O | O | O | O | O | O |
|  | Both models  involved | O | O | O | O | O | O | O | O | O |
| Skill  paradigm | Bilateral  access | O | O | O | O | O | O | O | O | O |
|  | Cross-  activation | O | O | O | O | O | O | O | O | O |
|  | Both models  involved | O | O | O | O | O | O | O | O | O |

Other comments (with reasons) _______________________________________________

6. Neuroanatomical evidence indicates that brain areas relating to the mirror neuron system (MNS) are activated when a unilateral motor task is performed and viewed with a mirror. In this light, a more recent hypothesis suggests that the transfer of strength and/or skills might be enhanced by observing our own motor action in a mirror during unimanual exercise, thereby activating the MNS.

Based on your knowledge, experience and experimental evidence, please judge the relevance of the MNS contribution to the phenomenon, distinguishing between the strength and skill paradigms.

|  | Not  relevant  at all |  |  |  | Neutral |  |  |  | Extremely  relevant |
| --- | --- | --- | --- | --- | --- | --- | --- | --- | --- |
| Strength  paradigm | O | O | O | O | O | O | O | O | O |
| Skill  paradigm | O | O | O | O | O | O | O | O | O |

Other comments (with reasons) _______________________________________________

7. Priming the ipsilateral M1 (i.e. using anodal tDCS prior to a single bout of strength exercise and motor skill practice) has been demonstrated to enhance the transfer phenomenon, providing support to the role of the ipsilateral M1 in regulating the transfer of performance.

Based on your knowledge, experience and experimental evidence, please judge the relevance of M1 priming contribution to the phenomenon.

|  | Not  relevant  at all |  |  |  | Neutral |  |  |  | Extremely  relevant |
| --- | --- | --- | --- | --- | --- | --- | --- | --- | --- |
| Strength  paradigm | O | O | O | O | O | O | O | O | O |
| Skill  paradigm | O | O | O | O | O | O | O | O | O |

Other comments (with reasons) _______________________________________________

8. Paired-pulse transcranial magnetic stimulation (TMS) is commonly used to study the function of the contralateral M1 following a session or a period of unilateral exercise, although characterization of many of the TMS-based ‘cortical’ parameters is not linked causatively with substantive improvements in motor function.

Based on your knowledge, experience and experimental evidence, please judge the importance of the following TMS-based outcomes to be included in the ideal neurophysiologic assessment of the phenomenon:

|  | Not an  important parameter to include |  |  |  | Neutral |  |  |  | A very  important parameter to include |
| --- | --- | --- | --- | --- | --- | --- | --- | --- | --- |
| 1 mV MEP | O | O | O | O | O | O | O | O | O |
| AMT | O | O | O | O | O | O | O | O | O |
| CMCT | O | O | O | O | O | O | O | O | O |
| CSP | O | O | O | O | O | O | O | O | O |
| ICF | O | O | O | O | O | O | O | O | O |
| LAI | O | O | O | O | O | O | O | O | O |
| LICI | O | O | O | O | O | O | O | O | O |
| LIHI | O | O | O | O | O | O | O | O | O |
| RC | O | O | O | O | O | O | O | O | O |
| RMT | O | O | O | O | O | O | O | O | O |
| SAI | O | O | O | O | O | O | O | O | O |
| SICF | O | O | O | O | O | O | O | O | O |
| SICI | O | O | O | O | O | O | O | O | O |
| SIHI | O | O | O | O | O | O | O | O | O |

*1 mV MEP, 1-millivolt motor evoked potential; AMT, active motor threshold; CMCT, central motor conduction time; CSP, cortical silent period; ICF, intracortical facilitation; LAI, long-latency afferent intra-cortical inhibition; LICI, long-interval intra-cortical inhibition; LIHI, long-latency interhemispheric inhibition; RC, recruitment curve; RMT, resting motor threshold; SAI, short-afferent intra-cortical inhibition; SICF, short-interval intracortical facilitation; SICI, short-interval intra-cortical inhibition; SIHI, short-latency interhemispheric inhibition.*

Other parameters to be considered______________________________________________

Other comments (with reasons) _______________________________________________

9. The contribution of muscular mechanisms to the phenomenon was apparently ruled out by early studies, which failed to detect morphological and enzymatic changes in the untrained muscles. These studies suffered, however, from potential technical limitations.

Is there merit in investigating the role of muscular mechanisms with new technologies?

| Not  worthy at all |  |  |  | Neutral |  |  |  | Definitely  worthy |
| --- | --- | --- | --- | --- | --- | --- | --- | --- |
| O | O | O | O | O | O | O | O | O |

Other comments (with reasons) _______________________________________________

10. Several mechanisms at different levels have been shown to be associated with the phenomenon, even though a cause-effect relationship with substantive improvements in motor function is lacking.

Based on your knowledge, experience and experimental evidence, please rank which mechanism is most likely (1) to least likely (8) to be associated with the phenomenon, distinguishing between the strength and skill paradigms. You can adjust the rank with the up/down arrows to the left of each option or drag/drop each option.

| Strength paradigm | | |  | Motor skill paradigm | | |
| --- | --- | --- | --- | --- | --- | --- |
| ⁞ | ↨ | Changes in voluntary activation |  | ⁞ | ↨ | Changes in voluntary activation |
| ⁞ | ↨ | Effect of remote contractions |  | ⁞ | ↨ | Effect of remote contractions |
| ⁞ | ↨ | Homeostatic plasticity |  | ⁞ | ↨ | Homeostatic plasticity |
| ⁞ | ↨ | Increased corticospinal  excitability |  | ⁞ | ↨ | Increased corticospinal  excitability |
| ⁞ | ↨ | Increased intracortical  facilitation |  | ⁞ | ↨ | Increased intracortical  facilitation |
| ⁞ | ↨ | Muscular mechanisms |  | ⁞ | ↨ | Muscular mechanisms |
| ⁞ | ↨ | New regions of cortical  activation |  | ⁞ | ↨ | New regions of cortical  activation |
| ⁞ | ↨ | Reduced interhemispheric  inhibition |  | ⁞ | ↨ | Reduced interhemispheric  inhibition |
| ⁞ | ↨ | Reduced intracortical inhibition |  | ⁞ | ↨ | Reduced intracortical inhibition |
| ⁞ | ↨ | Spinal reflex plasticity |  | ⁞ | ↨ | Spinal reflex plasticity |

Other mechanisms to be considered ______________________________________________

Other comments (with reasons) _______________________________________________

11. Besides muscle strength measurement, a number of techniques are currently employed to document the adaptations in response to unilateral training.

Based on your knowledge, experience and experimental evidence, please rank which technique is most likely (1) to least likely (6) to capture the adaptations. You can adjust the rank with the up/down arrows to the left of each option or drag/drop each option.

| ⁞ | ↨ | Anatomical magnetic resonance imaging |
| --- | --- | --- |
| ⁞ | ↨ | Muscle biopsy |
| ⁞ | ↨ | Electroencephalography |
| ⁞ | ↨ | EMG-based spinal reflex measurement |
| ⁞ | ↨ | Functional magnetic resonance imaging |
| ⁞ | ↨ | Positron emission tomography |
| ⁞ | ↨ | Structural neuroimaging  (diffusion tensor imaging) |
| ⁞ | ↨ | Transcranial magnetic stimulation  at the cortical, spinal and brainstem levels |
| ⁞ | ↨ | Twitch interpolation technique |

Other techniques _______________________________________________

Other comments (with reasons) _______________________________________________

12. There is experimental evidence suggesting that regions outside the primary motor cortex, but functionally connected to it, are activated during a unilateral motor task.

Based on your knowledge, experience and experimental evidence, please rank which CNS site is most likely (1) to least likely (9) to mediate/contribute to the phenomenon. You can adjust the rank with the up/down arrows to the left of each option or drag/drop each option.

| ⁞ | ↨ | Brainstem |
| --- | --- | --- |
| ⁞ | ↨ | Cerebellum |
| ⁞ | ↨ | Cingulate motor area |
| ⁞ | ↨ | Dorsal premotor cortex |
| ⁞ | ↨ | Primary motor cortex |
| ⁞ | ↨ | Primary somatosensory area |
| ⁞ | ↨ | Secondary somatosensory area |
| ⁞ | ↨ | Spinal cord |
| ⁞ | ↨ | Supplementary motor area |
| ⁞ | ↨ | Temporal lobe |

Other sites______________________________________________________

Other comments (with reasons) _______________________________________________

13. There is experimental evidence that the transfer of strength and/or skills can be maximized through specific strategies.

Based on your knowledge, experience and experimental evidence, please judge the potential value of the following strategies to enhance the transfer:

|  | Not  promising  at all |  |  |  | Neutral |  |  |  | Highly  promising |
| --- | --- | --- | --- | --- | --- | --- | --- | --- | --- |
| Eccentric  actions | O | O | O | O | O | O | O | O | O |
| High-intensity  training | O | O | O | O | O | O | O | O | O |
| Mirror  illusion | O | O | O | O | O | O | O | O | O |
| Motor  imagery | O | O | O | O | O | O | O | O | O |
| Peripheral  electrical  stimulation | O | O | O | O | O | O | O | O | O |
| Transcranial  electrical  stimulation | O | O | O | O | O | O | O | O | O |
| Vibration | O | O | O | O | O | O | O | O | O |

Other strategies to be considered______________________________________________

Other comments (with reasons) _______________________________________________

14. Experimental evidence from studies employing unilateral exercise paradigms on hand muscles has shown that the direction of the transfer (i.e. dominant to non-dominant, or vice versa) varies depending on the type of training (i.e. strength versus motor skill training).

Based on your knowledge, experience and experimental evidence, please state your degree of agreement with this position.

|  | Strongly  disagree |  |  |  | Neutral |  |  |  | Definitely  agree |
| --- | --- | --- | --- | --- | --- | --- | --- | --- | --- |
| For strength,  dominant to  non-dominant is  most pronounced | O | O | O | O | O | O | O | O | O |
| For strength,  non-dominant to  dominant is  most pronounced | O | O | O | O | O | O | O | O | O |
| For skills,  dominant to  non-dominant is  most pronounced | O | O | O | O | O | O | O | O | O |
| For skills,  non-dominant to  dominant is  most pronounced | O | O | O | O | O | O | O | O | O |

Other comments (with reasons) _______________________________________________

15. Although to a lesser degree than the hand, dominance can be determined also for the lower limb. However, a dominant-to-non-dominant direction is not commonly reported.

Based on your knowledge, experience and experimental evidence, please judge if future investigations on this topic are needed.

|  | Not  at all |  |  |  | Neutral |  |  |  | Definitely  yes |
| --- | --- | --- | --- | --- | --- | --- | --- | --- | --- |
| For studies on  strength transfer | O | O | O | O | O | O | O | O | O |
| For studies on  skill transfer | O | O | O | O | O | O | O | O | O |

Other comments (with reasons) _______________________________________________

16. There is high heterogeneity among the studies about the duration of unilateral exercise protocols and this makes it difficult to outline a reliable dose-response relationship.

Based on your knowledge, experience and experimental evidence, and excluding single-session acute studies, please judge the least dose of training sessions* to obtain significant contralateral gains:

|  |  | Not  adequate  at all |  |  |  | Neutral |  |  |  | Very  adequate |
| --- | --- | --- | --- | --- | --- | --- | --- | --- | --- | --- |
| Strength training | < 6 sessions | O | O | O | O | O | O | O | O | O |
|  | 7-12 sessions | O | O | O | O | O | O | O | O | O |
|  | 15-24 sessions | O | O | O | O | O | O | O | O | O |
|  | 27-36 sessions | O | O | O | O | O | O | O | O | O |
|  | >36 sessions | O | O | O | O | O | O | O | O | O |
| Skill training | < 6 sessions | O | O | O | O | O | O | O | O | O |
|  | 7-12 sessions | O | O | O | O | O | O | O | O | O |
|  | 15-24 sessions | O | O | O | O | O | O | O | O | O |
|  | 27-36 sessions | O | O | O | O | O | O | O | O | O |
|  | >36 sessions | O | O | O | O | O | O | O | O | O |

** Considering 3 sessions/week*

Other timeframes (with reasons) ____________________________________________________________

Other comments (with reasons) _______________________________________________

17. Very few studies have investigated the time-course of the phenomenon. However, defining this feature would inform decision-making on the appropriate duration of unilateral training protocols.

Based on your knowledge, experience and experimental evidence, please judge if future investigations on this topic are worthy.

| Not  worthy  at all |  |  |  | Neutral |  |  |  | Definitely  worthy |
| --- | --- | --- | --- | --- | --- | --- | --- | --- |
| O | O | O | O | O | O | O | O | O |

Other comments (with reasons) _______________________________________________

18. By current definition, the transfer of muscle strength is frequently investigated and quantified in studies on the contralateral effects of unilateral training. Given the well-known difference in strength between men and women, should studies on unilateral strength training take report men’s and women’s data separately? Secondly, should this be done also in studies on motor skill training/transfer?

|  | Absolutely  not |  |  |  | Neutral |  |  |  | Definitely  yes |
| --- | --- | --- | --- | --- | --- | --- | --- | --- | --- |
| Strength  studies | O | O | O | O | O | O | O | O | O |
| Skill  studies | O | O | O | O | O | O | O | O | O |

Other comments (with reasons) _______________________________________________

19. Based on your knowledge, experience and experimental evidence, please judge whether unilateral exercise of the sound or less-affected side may have clinical utility:

|  | Absolutely  not |  |  |  | Neutral |  |  |  | Definitely  yes |
| --- | --- | --- | --- | --- | --- | --- | --- | --- | --- |
| To increase  strength in  the affected  limb | O | O | O | O | O | O | O | O | O |
| To increase  motor skills  in the  affected  limb | O | O | O | O | O | O | O | O | O |

Other comments (with reasons) ____________________________________________________________

20. Contralateral training (i.e. training the sound or least-affected limb to obtain crossed motor improvements in the untrained, most-affected side) has been advocated – although often in the absence of robust experimental evidence – for the management of unilateral motor impairment of different pathological origin.

Based on your knowledge, experience and experimental evidence, please judge the potential of the phenomenon for each of the following clinical scenarios:

|  | Not  promising  at all |  |  |  | Neutral |  |  |  | Very  promising |
| --- | --- | --- | --- | --- | --- | --- | --- | --- | --- |
| Central  neurological  conditions | O | O | O | O | O | O | O | O | O |
| Orthopedic  conditions | O | O | O | O | O | O | O | O | O |
| Peripheral  neurological  conditions | O | O | O | O | O | O | O | O | O |
| Rheumatologic  conditions | O | O | O | O | O | O | O | O | O |
| Sports  injuries | O | O | O | O | O | O | O | O | O |

Reasons behind your judgements________________________________________________

Other scenarios/conditions to be considered (with reasons) _______________________________________________

Other comments (with reasons) _______________________________________________

21. Previous and recent meta-analyses report most pronounced transfer effect in the lower than upper limbs of healthy subjects. In a translational perspective, would you expect unilateral impairments of the lower limb to benefit more than the upper limb from unilateral contralateral training?

| Absolutely no |  |  |  | Neutral |  |  |  | Definitely  yes |
| --- | --- | --- | --- | --- | --- | --- | --- | --- |
| O | O | O | O | O | O | O | O | O |

Other comments (with reasons) _______________________________________________

22. Currently, the phenomenon appears of more relevance to researchers and is not commonly used by clinicians.

Based on your knowledge, experience and experimental evidence, please judge the relevance of the following elements as potentials barriers to the clinical employment of unilateral contralateral training:

|  | Not a  relevant  element |  |  |  | Neutral |  |  |  | A very  relevant  element |
| --- | --- | --- | --- | --- | --- | --- | --- | --- | --- |
| Absence of  conditioning  stimuli on the  weaker side^#^ | O | O | O | O | O | O | O | O | O |
| Apparent lack  of noticeable  changes in  muscle bulk | O | O | O | O | O | O | O | O | O |
| Inadequate  scholars’ and  clinicians’  education/  training | O | O | O | O | O | O | O | O | O |
| Lack of studies  assessing the  clinical  importance and  meaningfulness^*^ | O | O | O | O | O | O | O | O | O |
| Patient’s  compliance  and acceptance  of a ‘paradoxical’ protocol | O | O | O | O | O | O | O | O | O |
| Relatively  small  magnitude  of effect | O | O | O | O | O | O | O | O | O |
| Unconventional  nature | O | O | O | O | O | O | O | O | O |

*^#^ i.e. in terms of change of metabolic, proprioceptive and visco-elastic properties; *i.e. minimal detectable change, minimal clinically important change.*

Other barriers to be considered (with reasons) ________________________________________________

Other comments (with reasons) _______________________________________________

23. Studies on constraint-induced movement therapy and direct training in case of unilateral impairment of neurological origin (mainly stroke) have warned against contralateral approaches as they may enhance the interhemispheric imbalance, also exacerbating strength and/or skill asymmetry.

Based on your knowledge, experience and experimental evidence, please state your degree of agreement with such warning.

|  | Strongly  disagree |  |  |  | Neutral |  |  |  | Definitely  agree |
| --- | --- | --- | --- | --- | --- | --- | --- | --- | --- |
| Unilateral  training of  the less-affected  side may enhance  inter-hemispheric  imbalance | O | O | O | O | O | O | O | O | O |
| Unilateral  training of  the less -affected  side may enhance muscle strength  asymmetry | O | O | O | O | O | O | O | O | O |
| Unilateral  training of  the less-affected side may enhance motor skill  asymmetry | O | O | O | O | O | O | O | O | O |
| Asymmetry is less important if there are benefits for  the more affected limb | O | O | O | O | O | O | O | O | O |

Other potential contraindications (with reasons) ___________________________________________________

Other comments (with reasons) _______________________________________________

24. Is there merit in developing a roadmap, namely a scoping review, to critically appraise the clinical potential of the phenomenon?

| Not  worthy at all |  |  |  | Neutral |  |  |  | Definitely  worthy |
| --- | --- | --- | --- | --- | --- | --- | --- | --- |
| O | O | O | O | O | O | O | O | O |

Other comments (with reasons) _______________________________________________

**Delphi process for cross-education**

**Round 2 of 2**

*If the content of some items falls outside your specific expertise, so that you would feel uncomfortable or inadequate to provide an answer, please leave the items unanswered (consensus will be calculated from responders). Please, also specify the reason/s for non-response in the comments below the item/s left blank.*

1. Cross-education (from now on: ‘the phenomenon’ or ‘unilateral training’ or ‘contralateral training’ or ‘unilateral contralateral training’) is generally defined as the increase in muscle strength and/or motor skills in the opposite, untrained limb following a period of unilateral exercise training.

Should the definition be updated or expanded?

| No update/expansion  is  required |  |  |  | Neutral |  |  |  | An  update/  expansion  is required |
| --- | --- | --- | --- | --- | --- | --- | --- | --- |
| O | O | O | O | O | O | O | O | O |

Other comments (with reasons) _______________________________________________

2. Based on your knowledge, experience and experimental evidence, please judge the importance of the following elements to be part of the definition, in case an update is deemed necessary:

|  | Not  important at all |  |  |  | Neutral |  |  |  | A very  important element |
| --- | --- | --- | --- | --- | --- | --- | --- | --- | --- |
| Homologous  muscles | O | O | O | O | O | O | O | O | O |
| Neural nature | O | O | O | O | O | O | O | O | O |
| Skill  transfer | O | O | O | O | O | O | O | O | O |
| Training-  specific | O | O | O | O | O | O | O | O | O |

Other elements to be considered______________________________________________

Other comments (with reasons) _______________________________________________

3. Please, rate the following terms to indicate the phenomenon.

|  | A terrible name |  |  |  | Neutral |  |  |  | A very  brilliant  name |
| --- | --- | --- | --- | --- | --- | --- | --- | --- | --- |
| Contralateral  effects | O | O | O | O | O | O | O | O | O |
| Contralateral  effects of  unilateral training |  |  |  |  |  |  |  |  |  |
| Interlimb  transfer | O | O | O | O | O | O | O | O | O |

Other suggested names_________________________________________

Other comments (with reasons) _______________________________________________

4. Two theoretical models involving neural plasticity of the brain have been proposed to explain the phenomenon:

(1) the “bilateral access” (aka ‘callosal access’), which involves the development of motor engrams following unilateral movement that can be accessed not only by the trained limb, but also by the untrained limb;

(2) the “cross-activation” hypothesis (‘spillover’), which is based on the concept of unilateral contractions being driven by bilateral cortical activity in both the contralateral and ipsilateral motor cortex, producing lasting neuroplasticity in both cortices;

Based on your knowledge, experience and experimental evidence, please state your degree of agreement with each model.

***You should now answer in the context of STRENGTH paradigms.***

|  | Strongly  disagree |  |  |  | Neutral |  |  |  | Definitely  Agree |
| --- | --- | --- | --- | --- | --- | --- | --- | --- | --- |
| Bilateral  access | O | O | O | O | O | O | O | O | O |
| Bilateral access and  cross-activation  both involved | O | O | O | O | O | O | O | O | O |

Other comments (with reasons) _______________________________________________

5. Two theoretical models involving neural plasticity of the brain have been proposed to explain the phenomenon:

(1) the “bilateral access” (aka ‘callosal access’), which involves the development of motor engrams following unilateral movement that can be accessed not only by the trained limb, but also by the untrained limb;

(2) the “cross-activation” hypothesis (‘spillover’), which is based on the concept of unilateral contractions being driven by bilateral cortical activity in both the contralateral and ipsilateral motor cortex, producing lasting neuroplasticity in both cortices;

Based on your knowledge, experience and experimental evidence, please state your degree of agreement with each model.

***You should now answer in the context of SKILL paradigms.***

|  | Strongly  disagree |  |  |  | Neutral |  |  |  | Definitely  Agree |
| --- | --- | --- | --- | --- | --- | --- | --- | --- | --- |
| Bilateral  access | O | O | O | O | O | O | O | O | O |
| Cross-activation | O | O | O | O | O | O | O | O | O |
| Both models  involved | O | O | O | O | O | O | O | O | O |

Other comments (with reasons) _______________________________________________

6. Neuroanatomical evidence indicates that brain areas relating to the mirror neuron system (MNS) are activated when a unilateral motor task is performed and viewed with a mirror. In this light, a more recent hypothesis suggests that the transfer of strength and/or skills might be enhanced by observing our own motor action in a mirror during unimanual exercise, thereby activating the MNS.

Based on your knowledge, experience and experimental evidence, please judge the relevance of the MNS contribution to the phenomenon.

***You should now answer in the context of STRENGTH paradigms.***

| Not  relevant  at all |  |  |  | Neutral |  |  |  | Extremely  relevant |
| --- | --- | --- | --- | --- | --- | --- | --- | --- |
| O | O | O | O | O | O | O | O | O |
| O | O | O | O | O | O | O | O | O |

Other comments (with reasons) _______________________________________________

7. Priming the ipsilateral M1 (i.e. using anodal tDCS prior to a single bout of strength exercise and motor skill practice) has been demonstrated to enhance the transfer phenomenon, providing support to the role of the ipsilateral M1 in regulating the transfer of performance.

Based on your knowledge, experience and experimental evidence, please judge the relevance of M1 priming contribution to the phenomenon.

|  | Not  relevant  at all |  |  |  | Neutral |  |  |  | Extremely  relevant |
| --- | --- | --- | --- | --- | --- | --- | --- | --- | --- |
| Strength  paradigm | O | O | O | O | O | O | O | O | O |
| Skill  paradigm | O | O | O | O | O | O | O | O | O |

Other comments (with reasons) _______________________________________________

8. Paired-pulse transcranial magnetic stimulation (TMS) is commonly used to study the function of the contralateral M1 following a session or a period of unilateral exercise, although characterization of many of the TMS-based ‘cortical’ parameters is not linked causatively with substantive improvements in motor function.

Based on your knowledge, experience and experimental evidence, please judge the importance of the following TMS-based outcomes to be included in the ideal neurophysiologic assessment of the phenomenon:

|  | Not an  important parameter to include |  |  |  | Neutral |  |  |  | A very  important parameter to include |
| --- | --- | --- | --- | --- | --- | --- | --- | --- | --- |
| CSP | O | O | O | O | O | O | O | O | O |
| ICF | O | O | O | O | O | O | O | O | O |
| IHI | O | O | O | O | O | O | O | O | O |

*CSP, cortical silent period; ICF, intracortical facilitation; IHI, interhemispheric inhibition.*

Other parameters to be considered______________________________________________

Other comments (with reasons) _______________________________________________

9. The contribution of muscular mechanisms to the phenomenon was apparently ruled out by early studies, which failed to detect morphological and enzymatic changes in the untrained muscles. These studies suffered, however, from potential technical limitations.

Is there merit in investigating the role of muscular mechanisms with new technologies?

| Not  worthy at all |  |  |  | Neutral |  |  |  | Definitely  worthy |
| --- | --- | --- | --- | --- | --- | --- | --- | --- |
| O | O | O | O | O | O | O | O | O |

Other comments (with reasons) _______________________________________________

10. There is experimental evidence that the transfer of strength and/or skills can be maximized through specific strategies.

Based on your knowledge, experience and experimental evidence, please judge the potential value of the following strategies to enhance the transfer:

|  | Not  promising  at all |  |  |  | Neutral |  |  |  | Highly  promising |
| --- | --- | --- | --- | --- | --- | --- | --- | --- | --- |
| Eccentric  actions | O | O | O | O | O | O | O | O | O |
| Mirror  illusion | O | O | O | O | O | O | O | O | O |
| Motor  imagery | O | O | O | O | O | O | O | O | O |

Other strategies to be considered______________________________________________

Other comments (with reasons) _______________________________________________

11. Experimental evidence from studies employing unilateral exercise paradigms on hand muscles has shown that the direction of the transfer (i.e. dominant to non-dominant, or vice versa) varies depending on the type of training (i.e. strength versus motor skill training).

Based on your knowledge, experience and experimental evidence, please state your degree of agreement with this position.

|  | Strongly  disagree |  |  |  | Neutral |  |  |  | Definitely  agree |
| --- | --- | --- | --- | --- | --- | --- | --- | --- | --- |
| For strength,  dominant to  non-dominant is  most pronounced | O | O | O | O | O | O | O | O | O |
| For skills,  dominant to  non-dominant is  most pronounced | O | O | O | O | O | O | O | O | O |

Other comments (with reasons) _______________________________________________

12. Although to a lesser degree than the hand, dominance can be determined also for the lower limb. However, a dominant-to-non-dominant direction is not commonly reported.

Based on your knowledge, experience and experimental evidence, please judge if future investigations on this topic are needed.

|  | Not  at all |  |  |  | Neutral |  |  |  | Definitely  yes |
| --- | --- | --- | --- | --- | --- | --- | --- | --- | --- |
| For studies on  strength transfer | O | O | O | O | O | O | O | O | O |
| For studies on  skill transfer | O | O | O | O | O | O | O | O | O |

Other comments (with reasons) _______________________________________________

13. There is high heterogeneity among the studies about the duration of unilateral exercise protocols and this makes it difficult to outline a reliable dose-response relationship.

Based on your knowledge, experience and experimental evidence, and excluding single-session acute studies, please judge the least dose of training sessions* to obtain significant contralateral gains.

***You should now answer in the context of STRENGTH paradigms.***

|  | Not  adequate  at all |  |  |  | Neutral |  |  |  | Very  adequate |
| --- | --- | --- | --- | --- | --- | --- | --- | --- | --- |
| 13-18 sessions | O | O | O | O | O | O | O | O | O |
| 19-24 sessions | O | O | O | O | O | O | O | O | O |
| 25-30 sessions | O | O | O | O | O | O | O | O | O |
| 31-36 sessions | O | O | O | O | O | O | O | O | O |
| >36 sessions | O | O | O | O | O | O | O | O | O |

** Considering 3 sessions/week*

Other timeframes (with reasons) ____________________________________________________________

Other comments (with reasons) _______________________________________________

14. There is high heterogeneity among the studies about the duration of unilateral exercise protocols and this makes it difficult to outline a reliable dose-response relationship.

Based on your knowledge, experience and experimental evidence, and excluding single-session acute studies, please judge the least dose of training sessions* to obtain significant contralateral gains.

***You should now answer in the context of SKILL paradigms.***

|  | Not  adequate  at all |  |  |  | Neutral |  |  |  | Very  adequate |
| --- | --- | --- | --- | --- | --- | --- | --- | --- | --- |
| 7-12 sessions | O | O | O | O | O | O | O | O | O |
| 13-18 sessions | O | O | O | O | O | O | O | O | O |
| 19-24 sessions | O | O | O | O | O | O | O | O | O |
| 25-30 sessions | O | O | O | O | O | O | O | O | O |
| 31-36 sessions | O | O | O | O | O | O | O | O | O |

** Considering 3 sessions/week*

Other timeframes (with reasons) ____________________________________________________________

Other comments (with reasons) _______________________________________________

15. By current definition, the transfer of muscle strength is frequently investigated and quantified in studies on the contralateral effects of unilateral training. Given the well-known difference in strength between men and women, should studies on unilateral strength training take report men’s and women’s data separately?

|  | Absolutely  not |  |  |  | Neutral |  |  |  | Definitely  yes |
| --- | --- | --- | --- | --- | --- | --- | --- | --- | --- |
| Strength  studies | O | O | O | O | O | O | O | O | O |

Other comments (with reasons) _______________________________________________

16. Contralateral training (i.e. training the sound or least-affected limb to obtain crossed motor improvements in the untrained, most-affected side) has been advocated – although often in the absence of robust experimental evidence – for the management of unilateral motor impairment of different pathological origin.

Based on your knowledge, experience and experimental evidence, please judge the potential of the phenomenon for each of the following clinical scenarios:

|  | Not  promising  at all |  |  |  | Neutral |  |  |  | Very  promising |
| --- | --- | --- | --- | --- | --- | --- | --- | --- | --- |
| Central  neurological  conditions | O | O | O | O | O | O | O | O | O |

Other scenarios/conditions to be considered (with reasons) _______________________________________________
