# Supplementary material for: Contralateral Effects of Unilateral Strength and Skill Training: Modified Delphi Consensus to Establish Key Aspects of Cross-Education
Source: Sports Med. 2020 Nov 11;51(1):11–20. doi: 10.1007/s40279-020-01377-7 (PMC7806569; doi:10.1007/s40279-020-01377-7)
Supplement: Supplementary file 2 — Supplementary file2 (PDF 3187 kb) [file 40279_2020_1377_MOESM2_ESM.pdf]

Contralateral effects of unilateral training: Delphi process

Monday, December 16, 2019

Powered by SurveyMonkey®

Round One – Response rate

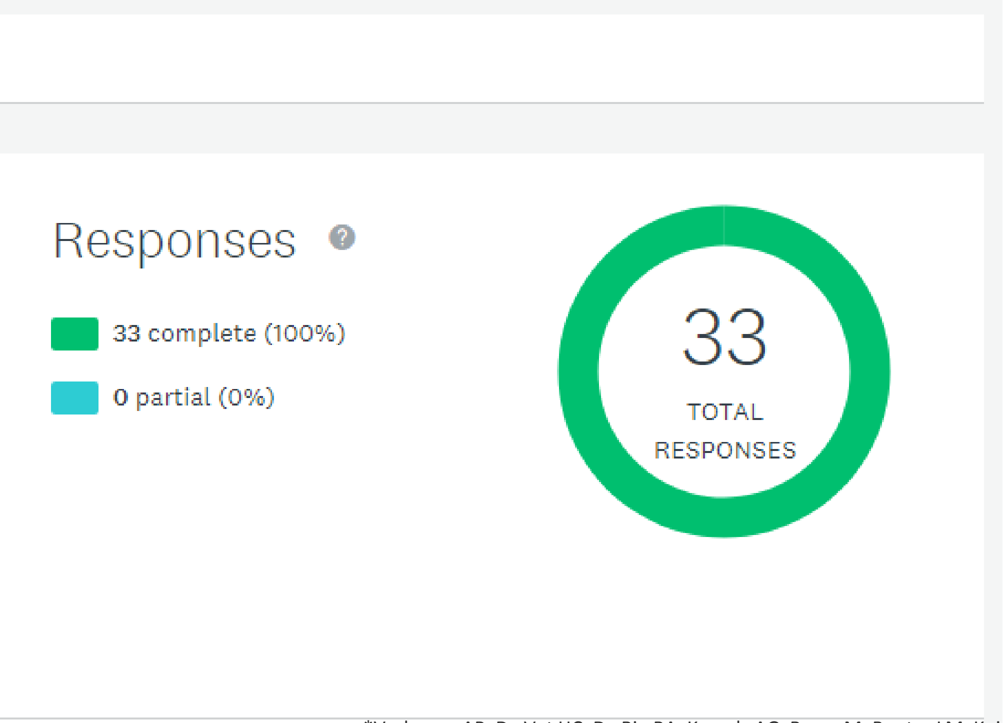

29 questions

Consensus set at 75%* #

*Verhagen AP, De Vet HC, De Bie RA, Kessels AG, Boers M, Bouter LM, Knipschild PG. The Delphi list: a criteria list for quality assessment of randomized clinical trials for conducting systematic reviews developed by Delphi consensus. *J Clin Epidemiol*. 1998;51(12):1235–41.

Powered by SurveyMonkey

Jünger S, Payne SA, Brine J, Radbruch L, Brearley SG. Guidance on Conducting and REporting DElphi Studies (CREDES) in palliative care: Recommendations based on a methodological systematic review. *Palliat Med*. 2017;31(8):684–706.

Delphi Process - Round 1 Summary Report

Thank you for participating in Round 1 of our Delphi Process to help aggregate opinions on a number of issues relating to contralateral training.

Summary of Round 1:

You will see that consensus (≥ 75%) was reached for 17 questions (green-boxed items) while not reached for 10 questions (red-boxed items). Of the latter, 4 items showed emerging support (between 70 and 74.9%). All items for which consensus was not obtained but reached ≥50% will be presented in Round 2. All items that reached less than 50% of preferences will not be represented in Round 2.

- **All responses from Round 1 are included in this document**

Round 2: In Round 2, we invite participants to consider the responses from other respondents, and to re-score items where consensus was not reached. Please use this document while re-scoring items in Round 2.

Powered by SurveyMonkey

Q1 Cross-education (from now on: ‘the phenomenon’ or ‘unilateral training’ or ‘contralateral training’ or ‘unilateral contralateral training’) is generally defined as the increase in muscle strength and/or motor skills in the opposite, untrained limb following a period of unilateral exercise training.

	NO UPDATE/EXPANSION IS REQUIRED	(NO LABEL)	NEUTRAL	(NO LABEL)	AN UPDATE/EXPANSION IS REQUIRED	TOTAL
Should the definition be updated or expanded?	15.63% 5	18.75% 6	12.50% 4	15.63% 5	37.50% 12	32

Consensus not reached (53.1%). Item is presented in Round 2.

Dotted line showing how the positive labels are aggregated into one percentage.

Powered by SurveyMonkey

Q2 **Based on your knowledge, experience and experimental evidence, please judge the importance of the following elements to be part of the definition, in case an update is deemed necessary:**

	NOT IMPORTANT AT ALL	(NO LABEL)	NEUTRAL	(NO LABEL)	A VERY IMPORTANT ELEMENT	TOTAL RESPONDENTS
Homologous muscles	0.00%<br>0	13.33%<br>4	13.33%<br>4	36.67%<br>11	36.67%<br>11	30
Intensity of the training	10.00%<br>3	30.00%<br>9	16.67%<br>5	26.67%<br>8	16.67%<br>5	30
Main putative neural mechanism	3.45%<br>1	13.79%<br>4	44.83%<br>13	20.69%<br>6	17.24%<br>5	29
Neural nature	6.67%<br>2	10.00%<br>3	23.33%<br>7	23.33%<br>7	36.67%<br>11	30
Skill transfer	3.23%<br>1	0.00%<br>0	25.81%<br>8	19.35%<br>6	51.61%<br>16	31
Strength transfer	3.23%<br>1	0.00%<br>0	16.13%<br>5	22.58%<br>7	58.06%<br>18	31
Training-specific	3.33%<br>1	3.33%<br>1	30.00%<br>9	36.67%<br>11	26.67%<br>8	30

Consensus reached on green-boxed items. These items removed from Round 2.
Red-boxed items (< 50%) removed from Round 2.
Four remaining items presented in Round 2.

Powered by SurveyMonkey

Q3 A considerable body of knowledge has accumulated so far on the phenomenon, i.e. contralateral effects of unilateral training (both strength and skill) and associated adaptations. Most researchers currently refer to this broad topic with the term 'cross-education'.

	NO CHANGE IN NAME REQUIRED	(NO LABEL)	NEUTRAL	(NO LABEL)	AN ALTERNATIVE NAME IS REQUIRED	TOTAL
Should this term be changed?	25.00% 8	6.25% 2	34.38% 11	9.38% 3	25.00% 8	32

Consensus not reached (34.4%). Item removed from Round 2.

Powered by SurveyMonkey

Q4 Please, rate the following terms to indicate the phenomenon.

	A TERRIBLE NAME	(NO LABEL)	NEUTRAL	(NO LABEL)	A VERY BRILLIANT NAME	TOTAL RESPONDENTS
Cross-education	0.00%<br>0	3.33%<br>1	16.67%<br>5	56.67%<br>17	23.33%<br>7	30
Contralateral effect	6.90%<br>2	34.48%<br>10	37.93%<br>11	13.79%<br>4	6.90%<br>2	29
Contralateral effects of unilateral training	3.23%<br>1	12.90%<br>4	19.35%<br>6	51.61%<br>16	12.90%<br>4	31
Contralateral training effect	10.00%<br>3	13.33%<br>4	16.67%<br>5	53.33%<br>16	6.67%<br>2	30
Cross-training	60.00%<br>18	20.00%<br>6	13.33%<br>4	6.67%<br>2	0.00%<br>0	30
Cross-training effect	58.06%<br>18	16.13%<br>5	12.90%<br>4	9.68%<br>3	3.23%<br>1	31
Cross-transfer	10.00%<br>3	20.00%<br>6	36.67%<br>11	26.67%<br>8	6.67%<br>2	30
Cross-transfer effect	9.68%<br>3	22.58%<br>7	29.03%<br>9	32.26%<br>10	6.45%<br>2	31
Interlimb transfer	0.00%<br>0	16.13%<br>5	32.26%<br>10	35.48%<br>11	16.13%<br>5	31

Consensus reached on green-boxed item. This item removed from Round 2.

Red-boxed items (< 50%) removed from Round 2.

Three remaining items presented in Round 2

Powered by SurveyMonkey

Q5 Two theoretical models involving neural plasticity of the brain have been proposed to explain the phenomenon: (1) the "bilateral access" (aka 'callosal access'), which involves the development of motor engrams following unilateral movement that can be accessed not only by the trained limb, but also by the untrained limb (2) the "cross-activation" hypothesis ('spillover'), which is based on the concept of unilateral contractions being driven by bilateral cortical activity in both the contralateral and ipsilateral motor cortex, producing lasting neuroplasticity in both cortices.
Based on your knowledge, experience and experimental evidence, please state your degree of agreement with each model. You should now answer in the context of STRENGTH paradigms.

	STRONGLY DISAGREE	(NO LABEL)	NEUTRAL	(NO LABEL)	DEFINITELY AGREE	TOTAL RESPONDENTS
Bilateral access	0.00%<br>0	7.69%<br>2	46.15%<br>12	38.46%<br>10	7.69%<br>2	26
Cross-activation	0.00%<br>0	3.85%<br>1	23.08%<br>6	38.46%<br>10	34.62%<br>9	26
Both models involved	0.00%<br>0	6.90%<br>2	24.14%<br>7	27.59%<br>8	41.38%<br>12	29

Red-boxed item (46.1 %) removed from Round 2.
Three remaining items presented in Round 2

Powered by SurveyMonkey

Q6 Two theoretical models involving neural plasticity of the brain have been proposed to explain the phenomenon: (1) the “bilateral access” (aka ‘callosal access’), which involves the development of motor engrams following unilateral movement that can be accessed not only by the trained limb, but also by the untrained limb (2) the “cross-activation” hypothesis (‘spillover’), which is based on the concept of unilateral contractions being driven by bilateral cortical activity in both the contralateral and ipsilateral motor cortex, producing lasting neuroplasticity in both cortices. Based on your knowledge, experience and experimental evidence, please state your degree of agreement with each model. You should now answer in the context of SKILL paradigms.

	STRONGLY DISAGREE	(NO LABEL)	NEUTRAL	(NO LABEL)	DEFINITELY AGREE	TOTAL RESPONDENTS
Bilateral access	0.00%<br>0	0.00%<br>0	42.31%<br>11	42.31%<br>11	15.38%<br>4	26
Cross-activation	0.00%<br>0	7.69%<br>2	38.46%<br>10	34.62%<br>9	19.23%<br>5	26
Both models involved	3.57%<br>1	0.00%<br>0	28.57%<br>8	39.29%<br>11	28.57%<br>8	28

Consensus not reached. Items are presented in Round 2.

Powered by SurveyMonkey

Q7 Neuroanatomical evidence indicates that brain areas relating to the mirror neuron system (MNS) are activated when a unilateral motor task is performed and viewed with a mirror. In this light, a more recent hypothesis suggests that the transfer of strength and/or skills might be enhanced by observing our own motor action in a mirror during unimanual exercise, thereby activating the MNS. Based on your knowledge, experience and experimental evidence, please judge the relevance of the MNS contribution to the phenomenon, distinguishing between the strength and skill paradigms.

	NOT RELEVANT AT ALL	(NO LABEL)	NEUTRAL	(NO LABEL)	EXTREMELY RELEVANT	TOTAL RESPONDENTS
Strength paradigm	0.00%<br>0	14.29%<br>4	35.71%<br>10	42.86%<br>12	10.71%<br>3	28
Skill paradigm	0.00%<br>0	3.57%<br>1	21.43%<br>6	39.29%<br>11	35.71%<br>10	28

Consensus reached on green-boxed item. This item removed from Round 2.

One remaining item presented in Round 2

Powered by SurveyMonkey

Q8 Priming the ipsilateral M1 (i.e. using anodal tDCS prior to a single bout of strength exercise or motor skill practice) has been demonstrated to enhance the transfer phenomenon, providing support to the role of the ipsilateral M1 in regulating the transfer of performance. Based on your knowledge, experience and experimental evidence, please judge the relevance of priming the M1 for the phenomenon.

	NOT RELEVANT AT ALL	(NO LABEL)	NEUTRAL	(NO LABEL)	EXTREMELY RELEVANT	TOTAL RESPONDENTS
Strength paradigm	6.90%<br>2	6.90%<br>2	31.03%<br>9	37.93%<br>11	17.24%<br>5	29
Skill paradigm	3.45%<br>1	6.90%<br>2	31.03%<br>9	31.03%<br>9	27.59%<br>8	29

Consensus not reached. Items are presented in Round 2.

Powered by SurveyMonkey

Q9 **Paired-pulse transcranial magnetic stimulation (TMS) is commonly used to study the function of the contralateral M1 following a session or a period of unilateral exercise. Based on your knowledge, experience and experimental evidence, please judge the importance of the following TMS-based outcomes to be included in the ideal neurophysiologic assessment of the phenomenon.**

	NOT AN IMPORTANT PARAMETER TO INCLUDE	(NO LABEL)	NEUTRAL	(NO LABEL)	A VERY IMPORTANT PARAMETER TO INCLUDE	TOTAL RESPONDENTS
1 mV MEP (1-millivolt motor evoked potential)	4.17% 1	20.83% 5	41.67% 10	16.67% 4	16.67% 4	24
AMT (active motor threshold)	4.00% 1	16.00% 4	36.00% 9	32.00% 8	12.00% 3	25
CMCT (central motor conduction time)	16.00% 4	24.00% 6	52.00% 13	8.00% 2	0.00% 0	25
CSP (cortical silent period)	0.00% 0	11.54% 3	19.23% 5	38.46% 10	30.77% 8	26
ICF (intracortical facilitation)	0.00% 0	7.69% 2	26.92% 7	46.15% 12	19.23% 5	26
LAI (long-latency afferent intra-cortical inhibition)	0.00% 0	11.54% 3	69.23% 18	11.54% 3	7.69% 2	26
LICI (long-interval intra-cortical inhibition)	3.85% 1	23.08% 6	34.62% 9	30.77% 8	7.69% 2	26
LIHI (long-latency interhemispheric inhibition)	0.00% 0	11.54% 3	57.69% 15	19.23% 5	11.54% 3	26
RC (recruitment curve)	3.85% 1	11.54% 3	30.77% 8	23.08% 6	30.77% 8	26
RMT (resting motor threshold)	3.85% 1	26.92% 7	42.31% 11	15.38% 4	15.38% 4	26
SAI (short-afferent intra-cortical inhibition)	0.00% 0	12.00% 3	60.00% 15	24.00% 6	4.00% 1	25
SICF (short-interval intracortical facilitation)	0.00% 0	7.69% 2	50.00% 13	34.62% 9	7.69% 2	26
SICI (short-interval intra-cortical inhibition(	0.00% 0	7.69% 2	11.54% 3	42.31% 11	38.46% 10	26
SIHI (short-latency interhemispheric inhibition)	0.00% 0	11.54% 3	42.31% 11	30.77% 8	15.38% 4	26

Consensus reached on green-boxed item.
This item removed from Round 2.
Red-boxed items (< 50%) removed from Round 2.
Three remaining items presented in Round 2

Powered by SurveyMonkey

Q10 The contribution of muscular mechanisms to the phenomenon was apparently ruled out by early studies, which failed to detect morphological and enzymatic changes in the untrained muscles. These studies suffered, however, from potential technical limitations.

	NOT WORTHY AT ALL	(NO LABEL)	NEUTRAL	(NO LABEL)	DEFINITELY WORTHY	TOTAL RESPONDENTS
Is there merit in investigating the role of muscular mechanisms with new technologies?	0.00%<br>0	20.00%<br>6	10.00%<br>3	23.33%<br>7	46.67%<br>14	30

Consensus not reached. Item is presented in Round 2.

Powered by SurveyMonkey

Q11 Several mechanisms at different levels have been shown to be associated with the phenomenon, even though a cause-effect relationship with substantive improvements in motor function is lacking. Based on your knowledge, experience and experimental evidence, please rank which mechanism is most likely (1) to least likely (8) to be associated with the phenomenon. You should now answer in the context of strength paradigms.

	1	2	3	4	5	6	7	8	9	10	TOTAL	SCORE
Reduced interhemispheric inhibition	25.93% 7	18.52% 5	18.52% 5	18.52% 5	7.41% 2	7.41% 2	3.70% 1	0.00% 0	0.00% 0	0.00% 0	27	8.00
Reduced intracortical inhibition	11.11% 3	33.33% 9	11.11% 3	14.81% 4	14.81% 4	3.70% 1	7.41% 2	3.70% 1	0.00% 0	0.00% 0	27	7.52
Increased corticospinal excitability	18.52% 5	14.81% 4	18.52% 5	7.41% 2	11.11% 3	11.11% 3	3.70% 1	7.41% 2	3.70% 1	3.70% 1	27	6.89
New regions of cortical activation	7.69% 2	19.23% 5	7.69% 2	15.38% 4	15.38% 4	11.54% 3	3.85% 1	7.69% 2	7.69% 2	3.85% 1	26	6.27
Changes in voluntary activation	18.52% 5	7.41% 2	7.41% 2	7.41% 2	11.11% 3	22.22% 6	3.70% 1	18.52% 5	0.00% 0	3.70% 1	27	6.15
Increased intracortical facilitation	3.85% 1	0.00% 0	15.38% 4	7.69% 2	26.92% 7	19.23% 5	11.54% 3	3.85% 1	7.69% 2	3.85% 1	26	5.50
Spinal reflex plasticity	7.41% 2	0.00% 0	14.81% 4	14.81% 4	3.70% 1	7.41% 2	22.22% 6	11.11% 3	11.11% 3	7.41% 2	27	5.07
Homeostatic plasticity	3.85% 1	7.69% 2	7.69% 2	7.69% 2	0.00% 0	3.85% 1	23.08% 6	19.23% 5	15.38% 4	11.54% 3	26	4.35
Effect of remote contractions	3.57% 1	3.57% 1	0.00% 0	3.57% 1	10.71% 3	3.57% 1	14.29% 4	14.29% 4	28.57% 8	17.86% 5	28	3.50
Muscular mechanisms	0.00% 0	0.00% 0	3.57% 1	3.57% 1	0.00% 0	7.14% 2	7.14% 2	14.29% 4	21.43% 6	42.86% 12	28	2.46

Consensus reached on the first 4 ranked positions. Question removed from Round 2.

Powered by SurveyMonkey

Q12 **Several mechanisms at different levels have been shown to be associated with the phenomenon, even though a cause-effect relationship with substantive improvements in motor function is lacking. Based on your knowledge, experience and experimental evidence, please rank which mechanism is most likely (1) to least likely (8) to be associated with the phenomenon. You should now answer in the context of skill paradigms.**

	1	2	3	4	5	6	7	8	9	10	TOTAL	SCORE
Reduced interhemispheric inhibition	16.67% 4	25.00% 6	16.67% 4	20.83% 5	4.17% 1	4.17% 1	4.17% 1	8.33% 2	0.00% 0	0.00% 0	24	7.58
New regions of cortical activation	23.08% 6	23.08% 6	11.54% 3	11.54% 3	7.69% 2	7.69% 2	7.69% 2	7.69% 2	0.00% 0	0.00% 0	26	7.50
Reduced intracortical inhibition	16.67% 4	25.00% 6	16.67% 4	20.83% 5	4.17% 1	0.00% 0	4.17% 1	4.17% 1	8.33% 2	0.00% 0	24	7.42
Increased corticospinal excitability	12.50% 3	12.50% 3	12.50% 3	16.67% 4	29.17% 7	16.67% 4	0.00% 0	0.00% 0	0.00% 0	0.00% 0	24	7.13
Increased intracortical facilitation	3.85% 1	0.00% 0	19.23% 5	7.69% 2	34.62% 9	19.23% 5	11.54% 3	3.85% 1	0.00% 0	0.00% 0	26	6.08
Homeostatic plasticity	17.39% 4	4.35% 1	13.04% 3	13.04% 3	8.70% 2	8.70% 2	13.04% 3	8.70% 2	8.70% 2	4.35% 1	23	6.04
Spinal reflex plasticity	4.00% 1	4.00% 1	8.00% 2	4.00% 1	4.00% 1	16.00% 4	28.00% 7	12.00% 3	16.00% 4	4.00% 1	25	4.56
Changes in voluntary activation	4.00% 1	8.00% 2	0.00% 0	4.00% 1	8.00% 2	8.00% 2	8.00% 2	32.00% 8	24.00% 6	4.00% 1	25	4.08
Effect of remote contractions	4.35% 1	4.35% 1	4.35% 1	4.35% 1	0.00% 0	8.70% 2	13.04% 3	21.74% 5	21.74% 5	17.39% 4	23	3.70
Muscular mechanisms	0.00% 0	0.00% 0	0.00% 0	0.00% 0	0.00% 0	4.17% 1	4.17% 1	4.17% 1	20.83% 5	66.67% 16	24	1.58

Consensus reached on the first 4 ranked positions. Question removed from Round 2.

Powered by SurveyMonkey

Q13 Besides muscle strength measurement, a number of other techniques are currently employed to document the adaptations in response to unilateral training. Based on your knowledge, experience and experimental evidence, please rank which technique is most likely (1) to least likely (6) to capture the adaptations.

	1	2	3	4	5	6	7	8	9	TOTAL	SCORE
Functional magnetic resonance imaging	32.00% 8	20.00% 5	32.00% 8	0.00% 0	16.00% 4	0.00% 0	0.00% 0	0.00% 0	0.00% 0	25	7.52
Transcranial magnetic stimulation at the cortical, spinal and brainstem levels	29.63% 8	18.52% 5	18.52% 5	0.00% 0	14.81% 4	7.41% 2	7.41% 2	3.70% 1	0.00% 0	27	6.78
Structural neuroimaging (diffusion tensor imaging)	17.39% 4	26.09% 6	8.70% 2	13.04% 3	0.00% 0	13.04% 3	13.04% 3	8.70% 2	0.00% 0	23	6.13
Positron emission tomography	0.00% 0	22.73% 5	9.09% 2	31.82% 7	4.55% 1	18.18% 4	9.09% 2	0.00% 0	4.55% 1	22	5.64
Electroencephalography	4.17% 1	8.33% 2	16.67% 4	16.67% 4	16.67% 4	20.83% 5	0.00% 0	4.17% 1	12.50% 3	24	5.08
EMG-based spinal reflex measurement	8.00% 2	4.00% 1	4.00% 1	20.00% 5	24.00% 6	16.00% 4	16.00% 4	8.00% 2	0.00% 0	25	5.00
Anatomical magnetic resonance imaging	9.52% 2	0.00% 0	9.52% 2	4.76% 1	9.52% 2	19.05% 4	19.05% 4	28.57% 6	0.00% 0	21	4.19
Twitch interpolation technique	3.85% 1	3.85% 1	3.85% 1	15.38% 4	7.69% 2	3.85% 1	15.38% 4	26.92% 7	19.23% 5	26	3.58
Muscle biopsy	0.00% 0	8.33% 2	0.00% 0	4.17% 1	0.00% 0	8.33% 2	12.50% 3	12.50% 3	54.17% 13	24	2.42

Consensus reached on the first 4 ranked positions. Question removed from Round 2.

Powered by SurveyMonkey

Q14 There is experimental evidence suggesting that regions outside the primary motor cortex, but functionally connected to it, are activated during a unilateral motor task. Based on your knowledge, experience and experimental evidence, please rank which CNS site is most likely (1) to least likely (9) to mediate/contribute to the phenomenon. You can adjust the rank with the up/down arrows to the left of each option or drag/drop each option.

	1	2	3	4	5	6	7	8	9	10	TOTAL	SCORE
Primary motor cortex	66.67% 16	8.33% 2	4.17% 1	4.17% 1	8.33% 2	4.17% 1	0.00% 0	4.17% 1	0.00% 0	0.00% 0	24	8.88
Supplementary motor area	4.17% 1	50.00% 12	4.17% 1	20.83% 5	8.33% 2	0.00% 0	0.00% 0	4.17% 1	4.17% 1	4.17% 1	24	7.46
Primary somatosensory area	8.70% 2	17.39% 4	21.74% 5	8.70% 2	17.39% 4	8.70% 2	8.70% 2	4.35% 1	4.35% 1	0.00% 0	23	6.83
Dorsal premotor cortex	0.00% 0	4.35% 1	17.39% 4	26.09% 6	30.43% 7	17.39% 4	0.00% 0	4.35% 1	0.00% 0	0.00% 0	23	6.43
Cerebellum	8.70% 2	4.35% 1	8.70% 2	8.70% 2	0.00% 0	30.43% 7	8.70% 2	13.04% 3	17.39% 4	0.00% 0	23	5.17
Secondary somatosensory area	0.00% 0	4.35% 1	21.74% 5	4.35% 1	8.70% 2	8.70% 2	30.43% 7	8.70% 2	8.70% 2	4.35% 1	23	5.09
Cingulate motor area	0.00% 0	8.70% 2	0.00% 0	13.04% 3	17.39% 4	13.04% 3	26.09% 6	4.35% 1	8.70% 2	8.70% 2	23	4.83
Spinal cord	4.17% 1	8.33% 2	4.17% 1	8.33% 2	4.17% 1	8.33% 2	4.17% 1	25.00% 6	8.33% 2	25.00% 6	24	4.08
Brainstem	8.70% 2	0.00% 0	8.70% 2	4.35% 1	4.35% 1	0.00% 0	13.04% 3	13.04% 3	30.43% 7	17.39% 4	23	3.83
Temporal lobe	0.00% 0	0.00% 0	8.70% 2	0.00% 0	0.00% 0	8.70% 2	8.70% 2	17.39% 4	17.39% 4	39.13% 9	23	2.74

Consensus reached on the first 4 ranked positions. Question removed from Round 2.

Powered by SurveyMonkey

Q15 There is experimental evidence that the transfer of strength and/or skills can be maximized through specific strategies. Based on your knowledge, experience and experimental evidence, please judge the potential value of the following strategies to enhance the transfer:

	NOT PROMISING AT ALL	(NO LABEL)	NEUTRAL	(NO LABEL)	HIGHLY PROMISING	TOTAL RESPONDENTS
Eccentric actions	3.45% 1	0.00% 0	27.59% 8	17.24% 5	51.72% 15	29
High-intensity training	0.00% 0	0.00% 0	10.00% 3	43.33% 13	46.67% 14	30
Mirror illusion	3.23% 1	0.00% 0	22.58% 7	51.61% 16	22.58% 7	31
Motor imagery	0.00% 0	13.33% 4	26.67% 8	43.33% 13	16.67% 5	30
Peripheral electrical stimulation	0.00% 0	13.33% 4	50.00% 15	26.67% 8	10.00% 3	30
Transcranial electrical stimulation	3.33% 1	10.00% 3	53.33% 16	30.00% 9	3.33% 1	30
Vibration	6.45% 2	29.03% 9	41.94% 13	22.58% 7	0.00% 0	31

Consensus reached on green-boxed item. This item removed from Round 2.
Red-boxed items (< 50%) removed from Round 2.
Three remaining items presented in Round 2.

Powered by SurveyMonkey

Q16 **Experimental evidence from studies employing unilateral exercise paradigms on hand muscles has shown that the direction of the transfer (i.e. dominant to non-dominant, or vice versa) varies depending on the type of training (i.e. strength versus motor skill training). Based on your knowledge, experience and experimental evidence, please state your degree of agreement with this position.**

	STRONGLY DISAGREE	(NO LABEL)	NEUTRAL	(NO LABEL)	DEFINITELY AGREE
For strength, dominant to non-dominant is most pronounced	0.00%<br>0	3.57%<br>1	46.43%<br>13	25.00%<br>7	25.00%<br>7
For strength, non-dominant to dominant is most pronounced	18.52%<br>5	22.22%<br>6	55.56%<br>15	3.70%<br>1	0.00%<br>0
For skills, dominant to non-dominant is most pronounced	0.00%<br>0	3.57%<br>1	50.00%<br>14	25.00%<br>7	25.00%<br>7
For skills, non-dominant to dominant is most pronounced	7.41%<br>2	33.33%<br>9	59.26%<br>16	0.00%<br>0	0.00%<br>0

Consensus not reached.
Red boxed items (< 50%) removed from Round 2.

Powered by SurveyMonkey

Q17 Although to a lesser degree than the hand, dominance can be determined also for the lower limb. However, a dominant-to-non-dominant direction is not commonly reported. Based on your knowledge, experience and experimental evidence, please judge if future investigations on this topic are needed.

	NOT NEEDED AT ALL	(NO LABEL)	NEUTRAL	(NO LABEL)	DEFINITELY NEEDED	TOTAL RESPONDENTS
For studies on strength transfer	3.57% 1	3.57% 1	25.00% 7	35.71% 10	32.14% 9	28
For studies on skill transfer	3.45% 1	3.45% 1	27.59% 8	37.93% 11	27.59% 8	29

Consensus not reached. Items are presented in Round 2.

Powered by SurveyMonkey

Q18 There is high heterogeneity among the studies about the duration of unilateral exercise protocols and this makes it difficult to outline a reliable dose-response relationship. Based on your knowledge, experience and experimental evidence, and excluding single-session acute studies, please judge the least dose of training sessions (considering a frequency of 3 sessions/week) to obtain significant contralateral gains. You should now answer in the context of strength paradigms.

	NOT ADEQUATE AT ALL	(NO LABEL)	NEUTRAL	(NO LABEL)	VERY ADEQUATE	TOTAL RESPONDENTS
<6 sessions	40.74% 11	18.52% 5	22.22% 6	14.81% 4	3.70% 1	27
7-12 sessions	8.00% 2	20.00% 5	24.00% 6	24.00% 6	24.00% 6	25
13-18 sessions	3.70% 1	0.00% 0	33.33% 9	25.93% 7	37.04% 10	27
19-24 sessions	0.00% 0	3.85% 1	26.92% 7	15.38% 4	53.85% 14	26
25-30 sessions	0.00% 0	0.00% 0	40.00% 10	12.00% 3	48.00% 12	25
31-36 sessions	0.00% 0	4.00% 1	40.00% 10	8.00% 2	48.00% 12	25
>36 sessions	0.00% 0	4.00% 1	40.00% 10	4.00% 1	52.00% 13	25

Red-boxed items (< 50%) removed from Round 2.

Five remaining items presented in Round 2.

Powered by SurveyMonkey

Q19 **There is high heterogeneity among the studies about the duration of unilateral exercise protocols and this makes it difficult to outline a reliable dose-response relationship. Based on your knowledge, experience and experimental evidence, and excluding single-session acute studies, please judge the least dose of training sessions (considering a frequency of 3 sessions/week) to obtain significant contralateral gains. You should now answer in the context of skill paradigms.**

	NOT ADEQUATE AT ALL	(NO LABEL)	NEUTRAL	(NO LABEL)	VERY ADEQUATE	TOTAL RESPONDENTS
<6 sessions	12.00% 3	12.00% 3	28.00% 7	28.00% 7	20.00% 5	25
7-12 sessions	8.00% 2	4.00% 1	32.00% 8	20.00% 5	36.00% 9	25
13-18 sessions	0.00% 0	8.00% 2	28.00% 7	24.00% 6	40.00% 10	25
19-24 sessions	0.00% 0	4.17% 1	29.17% 7	12.50% 3	54.17% 13	24
25-30 sessions	4.17% 1	4.17% 1	29.17% 7	8.33% 2	54.17% 13	24
31-36 sessions	8.33% 2	0.00% 0	33.33% 8	8.33% 2	50.00% 12	24
>36 sessions	8.70% 2	0.00% 0	34.78% 8	4.35% 1	52.17% 12	23

Red-boxed item (< 50%) removed from Round 2.

Six remaining items presented in Round 2.

Powered by SurveyMonkey

Q20 Very few studies have investigated the time-course of the phenomenon. However, defining this feature would inform decision-making on the appropriate duration of unilateral training protocols.

	NOT WORTHY AT ALL	(NO LABEL)	NEUTRAL	(NO LABEL)	DEFINITELY WORTHY	TOTAL
Are future investigations on the time-course of the crossed adaptations worthy of being conducted?	0.00%<br>0	6.90%<br>2	3.45%<br>1	20.69%<br>6	68.97%<br>20	29

Consensus reached. This question removed form Round 2.

Powered by SurveyMonkey

Q21 By current definition, the transfer of muscle strength is frequently investigated and quantified in studies on the contralateral effects of unilateral training. Given the well-known difference in strength between men and women, should studies on unilateral strength training report and analyze men's and women's data separately? Secondly, should this be done also in studies on motor skill transfer?

	ABSOLUTELY NOT	(NO LABEL)	NEUTRAL	(NO LABEL)	DEFINITELY YES	TOTAL RESPONDENTS
Strength studies	0.00%<br>0	13.79%<br>4	17.24%<br>5	24.14%<br>7	44.83%<br>13	29
Skill studies	3.33%<br>1	23.33%<br>7	30.00%<br>9	23.33%<br>7	20.00%<br>6	30

Red-boxed item (43.3%) is removed from Round 2.

Remaining item is presented in Round 2.

Q22 Based on your knowledge, experience and experimental evidence, please judge whether unilateral exercise of the sound/less-affected side may have clinical utility.

	ABSOLUTELY NOT	(NO LABEL)	NEUTRAL	(NO LABEL)	DEFINITELY YES	TOTAL RESPONDENTS
To increase strength in the affected limb	0.00%<br>0	3.23%<br>1	6.45%<br>2	25.81%<br>8	64.52%<br>20	31
To increase motor skills in the affected limb	0.00%<br>0	0.00%<br>0	3.23%<br>1	25.81%<br>8	70.97%<br>22	31

Consensus reached. This question removed from Round 2.

Powered by SurveyMonkey

Q23 Contralateral training (i.e. training the sound/less-affected limb to obtain crossed motor improvements in the untrained, most-affected side) has been advocated – although often in the absence of robust experimental evidence – for the management of unilateral motor impairment of different pathological origin. Based on your knowledge, experience and experimental evidence, please judge the potential of the phenomenon for each of the following clinical scenarios.

	NOT PROMISING AT ALL	(NO LABEL)	NEUTRAL	(NO LABEL)	VERY PROMISING	TOTAL RESPONDENTS
Central neurological conditions	0.00%<br>0	3.45%<br>1	37.93%<br>11	24.14%<br>7	34.48%<br>10	29
Orthopedic conditions	0.00%<br>0	10.71%<br>3	7.14%<br>2	42.86%<br>12	39.29%<br>11	28
Peripheral neurological conditions	7.14%<br>2	14.29%<br>4	32.14%<br>9	35.71%<br>10	10.71%<br>3	28
Rheumatologic conditions	14.29%<br>4	17.86%<br>5	42.86%<br>12	17.86%<br>5	7.14%<br>2	28
Sports injuries	0.00%<br>0	3.33%<br>1	3.33%<br>1	53.33%<br>16	40.00%<br>12	30

Consensus reached on green-boxed items. These items removed from Round 2.
Red-boxed items (< 50%) removed from Round 2.
One remaining item presented in Round 2.

Powered by SurveyMonkey

Q24 Previous and recent meta-analyses report most pronounced transfer effect in the lower than upper limbs of healthy subjects.

	ABSOLUTELY NOT	(NO LABEL)	NEUTRAL	(NO LABEL)	DEFINITELY YES	TOTAL
In a translational perspective, would you expect unilateral impairments of the lower limb to benefit more than the upper limb from unilateral contralateral training?	10.34%<br>3	20.69%<br>6	44.83%<br>13	17.24%<br>5	6.90%<br>2	29

Consensus not reached

Consensus not reached (24.1%). Item removed from Round 2.

Powered by SurveyMonkey

Q25 Currently, the phenomenon appears of more relevance to researchers and is not commonly used by clinicians. Based on your knowledge, experience and experimental evidence, please judge the relevance of the following elements as potentials barriers to the clinical employment of unilateral contralateral training.

Consensus reached on green-boxed items. These items removed from Round 2.

Red-boxed items (< 50%) removed from Round 2.

	NOT A RELEVANT BARRIER	(NO LABEL)	NEUTRAL	(NO LABEL)	A VERY RELEVANT BARRIER	TOTAL RESPONDENTS
Absence of conditioning stimuli on the weaker side (i.e. changes in metabolic, proprioceptive and visco-elastic properties)	16.67% 5	10.00% 3	43.33% 13	20.00% 6	10.00% 3	30
Apparent lack of noticeable changes in muscle bulk	26.67% 8	23.33% 7	26.67% 8	23.33% 7	0.00% 0	30
Inadequate scholars' and clinicians' education/training	3.33% 1	6.67% 2	13.33% 4	23.33% 7	53.33% 16	30
Lack of studies assessing the clinical importance and meaningfulness of the crossed change achieved (i.e. minimal detectable change, minimal clinically important change)	3.33% 1	6.67% 2	3.33% 1	23.33% 7	63.33% 19	30
Patient's compliance and acceptance of a 'paradoxical' protocol	3.33% 1	16.67% 5	46.67% 14	20.00% 6	13.33% 4	30
Relatively small magnitude of effect	0.00% 0	23.33% 7	40.00% 12	23.33% 7	13.33% 4	30
Unconventional nature	10.00% 3	13.33% 4	30.00% 9	23.33% 7	23.33% 7	30

Powered by SurveyMonkey

Q26 **Studies on constraint-induced movement therapy and direct training in case of unilateral impairment of neurological origin (mainly stroke) have warned against contralateral approaches as they may enhance the interhemispheric imbalance, also exacerbating strength and/or skill asymmetry. Based on your knowledge, experience and experimental evidence, please state your degree of agreement with such warning.**

	STRONGLY DISAGREE	(NO LABEL)	NEUTRAL	(NO LABEL)	DEFINITELY AGREE
Unilateral training of the less-affected side may enhance inter-hemispheric imbalance	12.00% 3	32.00% 8	40.00% 10	8.00% 2	8.00% 2
Unilateral training of the less - affected side may enhance muscle strength asymmetry	0.00% 0	32.00% 8	44.00% 11	20.00% 5	4.00% 1
Unilateral training of the less-affected side may enhance motor skill asymmetry	4.00% 1	32.00% 8	24.00% 6	28.00% 7	12.00% 3
Asymmetry is less important if there are benefits for the more affected limb	0.00% 0	8.00% 2	16.00% 4	40.00% 10	36.00% 9

Consensus reached on green-boxed item.
This item removed from Round 2.
Red-boxed items (< 50%) removed from Round 2.

Powered by SurveyMonkey

Q27 A scoping review is defined as a type of research synthesis that aims to 'map' the literature on a particular topic or research area and provides an opportunity to identify key concepts; gaps in the research; and types and sources of evidence to inform practice, policymaking, and research.

	NOT WORTHY AT ALL	(NO LABEL)	NEUTRAL	(NO LABEL)	DEFINITELY WORTHY	TOTAL
Is there merit in developing a scoping review to critically appraise the clinical potential of the phenomenon?	3.13%<br>1	0.00%<br>0	9.38%<br>3	31.25%<br>10	56.25%<br>18	32

Consensus reached. This question removed from Round 2.

Powered by SurveyMonkey

Q28 Thinking about your research activity, which of the following best describes you?

	STRONGLY DISAGREE	(NO LABEL)	NEUTRAL	(NO LABEL)	DEFINITELY AGREE	TOTAL RESPONDENTS
Researcher or Professor (basic science)	7.69% 2	7.69% 2	19.23% 5	11.54% 3	53.85% 14	26
Researcher or Professor (applied science)	7.14% 2	0.00% 0	0.00% 0	35.71% 10	57.14% 16	28
Not a researcher/professor	87.50% 14	0.00% 0	6.25% 1	6.25% 1	0.00% 0	16

Item not for consensus

Q29 Which of the following educational backgrounds best describes your professional figure?

ANSWER CHOICES	RESPONSES	
Sport scientist	53.13%	17
Other (please specify)	18.75%	6
Physiotherapist	12.50%	4
Biologist	6.25%	2
Medical doctor	6.25%	2
Bioengineer	3.13%	1
Nurse	0.00%	0
TOTAL		32

Item not for consensus
