# Supplementary material for: Contralateral Effects of Unilateral Strength and Skill Training: Modified Delphi Consensus to Establish Key Aspects of Cross-Education
Source: Sports Med. 2020 Nov 11;51(1):11–20. doi: 10.1007/s40279-020-01377-7 (PMC7806569; doi:10.1007/s40279-020-01377-7)
Supplement: Supplementary file 3 — Supplementary file3 (PDF 609 kb) [file 40279_2020_1377_MOESM3_ESM.pdf]

Round Two - Contralateral effects of unilateral training: Delphi process

Friday, February 28, 2020

Powered by SurveyMonkey®

32

Total Responses

Date Created: Wednesday, January 22, 2020

Complete Responses: 32

Q1: Cross-education (from now on: ‘the phenomenon’ or ‘unilateral training’ or ‘contralateral training’ or ‘unilateral contralateral training’) is generally defined as the increase in muscle strength and/or motor skills in the opposite, untrained limb following a period of unilateral exercise training.

Answered: 32   Skipped: 0

	NO UPDATE/EXPANSION IS REQUIRED	(NO LABEL)	NEUTRAL	(NO LABEL)	AN UPDATE/EXPANSION IS REQUIRED
Should the definition be updated or expanded?	18.75%<br>6	25.00%<br>8	9.38%<br>3	21.88%<br>7	25.00%<br>8

Powered by SurveyMonkey

Q2: Based on your knowledge, experience and experimental evidence, please judge the importance of the following elements to be part of the definition, in case an update is deemed necessary:

Answered: 31   Skipped: 1

	NOT IMPORTANT AT ALL	(NO LABEL)	NEUTRAL	(NO LABEL)	A VERY IMPORTANT ELEMENT	TOTAL
Homologous muscles	3.33%<br>1	3.33%<br>1	13.33%<br>4	33.33%<br>10	46.67%<br>14	30
Neural nature	6.67%<br>2	13.33%<br>4	26.67%<br>8	6.67%<br>2	46.67%<br>14	30
Skill transfer	3.33%<br>1	10.00%<br>3	26.67%<br>8	13.33%<br>4	46.67%<br>14	30
Training-specific	3.23%<br>1	6.45%<br>2	19.35%<br>6	38.71%<br>12	32.26%<br>10	31

Consensus reached

Powered by SurveyMonkey

Q3: Please, rate the following terms to indicate the phenomenon.

Answered: 32   Skipped: 0

	A TERRIBLE NAME	(NO LABEL)	NEUTRAL	(NO LABEL)	A VERY BRILLIANT NAME	TOTAL
Contralateral effects of unilateral training	0.00%<br>0	6.25%<br>2	31.25%<br>10	43.75%<br>14	18.75%<br>6	32
Contralateral training effect	6.25%<br>2	15.63%<br>5	28.13%<br>9	37.50%<br>12	12.50%<br>4	32
Interlimb transfer	18.75%<br>6	12.50%<br>4	21.88%<br>7	31.25%<br>10	15.63%<br>5	32

Powered by SurveyMonkey

Q4: Two theoretical models involving neural plasticity of the brain have been proposed to explain the phenomenon: (1) the “bilateral access” (aka ‘callosal access’), which involves the development of motor engrams following unilateral movement that can be accessed not only by the trained limb, but also by the untrained limb (2) the “cross-activation” hypothesis (‘spillover’), which is based on the concept of unilateral contractions being driven by bilateral cortical activity in both the contralateral and ipsilateral motor cortex, producing lasting neuroplasticity in both corticesBased on your knowledge, experience and experimental evidence, please state your degree of agreement with each model. You should now answer in the context of STRENGTH paradigms.

Answered: 32 Skipped: 0

	STRONGLY DISAGREE	(NO LABEL)	NEUTRAL	(NO LABEL)	DEFINITELY AGREE	TOTAL
Cross-activation	0.00%<br>0	0.00%<br>0	35.48%<br>11	35.48%<br>11	29.03%<br>9	31
Bilateral access and cross-activation both involved	3.13%<br>1	0.00%<br>0	28.13%<br>9	31.25%<br>10	37.50%<br>12	32

Powered by SurveyMonkey

Q5: Two theoretical models involving neural plasticity of the brain have been proposed to explain the phenomenon: (1) the “bilateral access” (aka ‘callosal access’), which involves the development of motor engrams following unilateral movement that can be accessed not only by the trained limb, but also by the untrained limb (2) the “cross-activation” hypothesis (‘spillover’), which is based on the concept of unilateral contractions being driven by bilateral cortical activity in both the contralateral and ipsilateral motor cortex, producing lasting neuroplasticity in both corticesBased on your knowledge, experience and experimental evidence, please state your degree of agreement with each model. You should now answer in the context of SKILL paradigms.

	STRONGLY DISAGREE	(NO LABEL)	NEUTRAL	(NO LABEL)	DEFINITELY AGREE	TOTAL
Bilateral access	0.00%<br>0	0.00%<br>0	27.59%<br>8	44.83%<br>13	27.59%<br>8	29
Cross-activation	0.00%<br>0	6.90%<br>2	51.72%<br>15	34.48%<br>10	6.90%<br>2	29
Both models involved	0.00%<br>0	0.00%<br>0	23.33%<br>7	36.67%<br>11	40.00%<br>12	30

Consensus reached

Powered by SurveyMonkey

Q6: Neuroanatomical evidence indicates that brain areas relating to the mirror neuron system (MNS) are activated when a unilateral motor task is performed and viewed with a mirror. In this light, a more recent hypothesis suggests that the transfer of strength and/or skills might be enhanced by observing our own motor action in a mirror during unimanual exercise, thereby activating the MNS. Based on your knowledge, experience and experimental evidence, please judge the relevance of the MNS contribution to the phenomenon, distinguishing between the strength and skill paradigms.

Answered: 31 Skipped: 1

	NOT RELEVANT AT ALL	(NO LABEL)	NEUTRAL	(NO LABEL)	EXTREMELY RELEVANT	TOTAL
Strength paradigm	0.00%<br>0	6.45%<br>2	32.26%<br>10	45.16%<br>14	16.13%<br>5	31

Powered by SurveyMonkey

Q7: Priming the ipsilateral M1 (i.e. using anodal tDCS prior to a single bout of strength exercise or motor skill practice) has been demonstrated to enhance the transfer phenomenon, providing support to the role of the ipsilateral M1 in regulating the transfer of performance.Based on your knowledge, experience and experimental evidence, please judge the relevance of priming the M1 for the phenomenon.

Answered: 30 Skipped: 2

	NOT RELEVANT AT ALL	(NO LABEL)	NEUTRAL	(NO LABEL)	EXTREMELY RELEVANT	TOTAL
Strength paradigm	0.00%<br>0	6.67%<br>2	36.67%<br>11	46.67%<br>14	10.00%<br>3	30
Skill paradigm	0.00%<br>0	3.33%<br>1	33.33%<br>10	56.67%<br>17	6.67%<br>2	30

Powered by SurveyMonkey

Q8: Paired-pulse transcranial magnetic stimulation (TMS) is commonly used to study the function of the contralateral M1 following a session or a period of unilateral exercise, although characterization of many of the TMS-based 'cortical' parameters is not linked causatively with substantive improvements in motor function.Based on your knowledge, experience and experimental evidence, please judge the importance of the following TMS-based outcomes to be included in the ideal neurophysiologic assessment of the phenomenon.

Answered: 29

	NOT AN IMPORTANT PARAMETER TO INCLUDE	(NO LABEL)	NEUTRAL	(NO LABEL)	A VERY IMPORTANT PARAMETER TO INCLUDE	TOTAL	
CSP (cortical silent period)	3.45%<br>1	6.90%<br>2	27.59%<br>8	44.83%<br>13	17.24%<br>5	29	
ICF (intracortical facilitation)	3.45%<br>1	13.79%<br>4	31.03%<br>9	44.83%<br>13	6.90%<br>2	29	
IHI (interhemispheric inhibition)	0.00%<br>0	3.45%<br>1	20.69%<br>6	34.48%<br>10	41.38%<br>12	29	Consensus reached
RC (recruitment curve)	3.45%<br>1	6.90%<br>2	51.72%<br>15	27.59%<br>8	10.34%<br>3	29	

Powered by SurveyMonkey

Q9: The contribution of muscular mechanisms to the phenomenon was apparently ruled out by early studies, which failed to detect morphological and enzymatic changes in the untrained muscles. These studies suffered, however, from potential technical limitations.

Answered: 32   Skipped: 0

	NOT WORTHY AT ALL	(NO LABEL)	NEUTRAL	(NO LABEL)	DEFINITELY WORTHY
Is there merit in investigating the role of muscular mechanisms with new technologies?	0.00%<br>0	6.45%<br>2	9.68%<br>3	29.03%<br>9	54.84%<br>17

Consensus reached

Q10: There is experimental evidence that the transfer of strength and/or skills can be maximized through specific strategies. Based on your knowledge, experience and experimental evidence, please judge the potential value of the following strategies to enhance the transfer:

Answered: 32 Skipped: 0

	NOT PROMISING AT ALL	(NO LABEL)	NEUTRAL	(NO LABEL)	HIGHLY PROMISING	TOTAL	
Eccentric actions	0.00%<br>0	3.13%<br>1	12.50%<br>4	34.38%<br>11	50.00%<br>16	32	Consensus reached
Mirror illusion	0.00%<br>0	3.13%<br>1	9.38%<br>3	53.13%<br>17	34.38%<br>11	32	Consensus reached
Motor imagery	0.00%<br>0	6.45%<br>2	35.48%<br>11	41.94%<br>13	16.13%<br>5	31	

Powered by SurveyMonkey

Q11: Experimental evidence from studies employing unilateral exercise paradigms on hand muscles has shown that the direction of the transfer (i.e. dominant to non-dominant, or vice versa) varies depending on the type of training (i.e. strength versus motor skill training). Based on your knowledge, experience and experimental evidence, please state your degree of agreement with this position.

Answered: 30 Skipped: 2

	STRONGLY DISAGREE	(NO LABEL)	NEUTRAL	(NO LABEL)	DEFINITELY AGREE	TOTAL
For strength, dominant to non-dominant is most pronounced	3.33%<br>1	13.33%<br>4	36.67%<br>11	30.00%<br>9	16.67%<br>5	30
For skills, dominant to non-dominant is most pronounced	3.45%<br>1	6.90%<br>2	62.07%<br>18	17.24%<br>5	10.34%<br>3	29

Powered by SurveyMonkey

Q12: Although to a lesser degree than the hand, dominance can be determined also for the lower limb. However, a dominant-to-non-dominant direction is not commonly reported. Based on your knowledge, experience and experimental evidence, please judge if future investigations on this topic are needed.

Answered: 30   Skipped: 2

	NOT NEEDED AT ALL	(NO LABEL)	NEUTRAL	(NO LABEL)	DEFINITELY NEEDED	TOTAL
For studies on strength transfer	0.00%<br>0	10.00%<br>3	26.67%<br>8	33.33%<br>10	30.00%<br>9	30
For studies on skill transfer	0.00%<br>0	13.33%<br>4	33.33%<br>10	30.00%<br>9	23.33%<br>7	30

Powered by SurveyMonkey

Q13: There is high heterogeneity among the studies about the duration of unilateral exercise protocols and this makes it difficult to outline a reliable dose-response relationship. Based on your knowledge, experience and experimental evidence, and excluding single-session acute studies, please judge the least dose of training sessions (considering a frequency of 3 sessions/week) to obtain significant contralateral gains.You should now answer in the context of strength paradigms.

Answered: 30 Skippe

	NOT ADEQUATE AT ALL	(NO LABEL)	NEUTRAL	(NO LABEL)	VERY ADEQUATE
13-18 sessions	3.45% 1	3.45% 1	17.24% 5	24.14% 7	51.72% 15
19-24 sessions	3.70% 1	3.70% 1	22.22% 6	18.52% 5	51.85% 14
25-30 sessions	0.00% 0	8.00% 2	52.00% 13	12.00% 3	28.00% 7
31-36 sessions	0.00% 0	11.54% 3	50.00% 13	3.85% 1	34.62% 9
>36 sessions	3.85% 1	15.38% 4	42.31% 11	3.85% 1	34.62% 9

Consensus reached

Powered by SurveyMonkey

Q14: There is high heterogeneity among the studies about the duration of unilateral exercise protocols and this makes it difficult to outline a reliable dose-response relationship. Based on your knowledge, experience and experimental evidence, and excluding single-session acute studies, please judge the least dose of training sessions (considering a frequency of 3 sessions/week) to obtain significant contralateral gains.You should now answer in the context of skill paradigms.

Answered: 29 Skipped: 3

	NOT ADEQUATE AT ALL	(NO LABEL)	NEUTRAL	(NO LABEL)	VERY ADEQUATE	TOTAL
7-12 sessions	7.14% 2	3.57% 1	21.43% 6	10.71% 3	57.14% 16	28
13-18 sessions	3.85% 1	7.69% 2	23.08% 6	19.23% 5	46.15% 12	26
19-24 sessions	0.00% 0	11.11% 3	25.93% 7	29.63% 8	33.33% 9	27
25-30 sessions	0.00% 0	7.69% 2	46.15% 12	15.38% 4	30.77% 8	26
31-36 sessions	0.00% 0	11.54% 3	46.15% 12	3.85% 1	38.46% 10	26
>36 sessions	3.85% 1	11.54% 3	46.15% 12	3.85% 1	34.62% 9	26

Powered by SurveyMonkey

Q15: By current definition, the transfer of muscle strength is frequently investigated and quantified in studies on the contralateral effects of unilateral training. Given the well-known difference in strength between men and women, should studies on unilateral strength training report and analyze men's and women's data separately? Secondly, should this be done also in studies on motor skill transfer?

Answered: 31   Skipped: 1

	ABSOLUTELY NOT	(NO LABEL)	NEUTRAL	(NO LABEL)	DEFINITELY YES	TOTAL
Strength studies	3.23%<br>1	16.13%<br>5	16.13%<br>5	19.35%<br>6	45.16%<br>14	31

Powered by SurveyMonkey

Q16: Contralateral training (i.e. training the sound/less-affected limb to obtain crossed motor improvements in the untrained, most-affected side) has been advocated – although often in the absence of robust experimental evidence – for the management of unilateral motor impairment of different pathological origin.Based on your knowledge, experience and experimental evidence, please judge the potential of the phenomenon for the following clinical scenario.

Answered: 30 Skipped: 2

	NOT PROMISING AT ALL	(NO LABEL)	NEUTRAL	(NO LABEL)	VERY PROMISING	TOTAL
Central neurological conditions	3.33%<br>1	3.33%<br>1	26.67%<br>8	36.67%<br>11	30.00%<br>9	30

Powered by SurveyMonkey

Q17: Thinking about your research activity, which of the following best describes you?

Answered: 30   Skipped: 2

	STRONGLY DISAGREE	(NO LABEL)	NEUTRAL	(NO LABEL)	DEFINITELY AGREE	TOTAL
Researcher or Professor (basic science)	4.55%<br>1	0.00%<br>0	18.18%<br>4	13.64%<br>3	63.64%<br>14	22
Researcher or Professor (applied science)	3.70%<br>1	0.00%<br>0	3.70%<br>1	22.22%<br>6	70.37%<br>19	27
Not a researcher/professor	100.00%<br>17	0.00%<br>0	0.00%<br>0	0.00%<br>0	0.00%<br>0	17

Powered by SurveyMonkey

Q18: Which of the following educational backgrounds best describes your professional figure?

Answered: 32   Skipped: 0

ANSWER CHOICES	RESPONSES	
Bioengineer	0.00%	0
Biologist	3.13%	1
Medical doctor	18.75%	6
Nurse	0.00%	0
Physiotherapist	15.63%	5
Sport scientist	56.25%	18
Other (please specify)	6.25%	2
TOTAL		32

Powered by SurveyMonkey
